# An automated microliter-scale high-throughput screening system (MSHTS) for real-time monitoring of protein aggregation using quantum-dot nanoprobes

**DOI:** 10.1038/s41598-019-38958-0

**Published:** 2019-02-22

**Authors:** Rina Sasaki, Reina Tainaka, Yuichi Ando, Yurika Hashi, Hadya V. Deepak, Yoshiko Suga, Yuta Murai, Masaki Anetai, Kenji Monde, Kiminori Ohta, Ikuko Ito, Haruhisa Kikuchi, Yoshiteru Oshima, Yasuyuki Endo, Hitomi Nakao, Masafumi Sakono, Koji Uwai, Kiyotaka Tokuraku

**Affiliations:** 1 Graduate School of Engineering, Muroran Institute of Technology, Muroran, Hokkaido Japan; 2 Yamano College of Aesthetics, Hachioji, Tokyo Japan; 3 Frontier Research Center for Advanced Material and Life Science, Faculty of Advanced Life Science, Hokkaido University, Sapporo, Hokkaido Japan; 4 Faculty of Pharmaceutical Sciences, Tohoku Medical and Pharmaceutical University, Sendai, Miyagi Japan; 5 School of Pharmacy, Showa University, Tokyo, Japan; 6 Graduate School of Pharmaceutical Sciences, Tohoku University, Sendai, Miyagi Japan; 7 Graduate School of Science and Engineering, University of Toyama, Toyama, Japan

## Abstract

Protein aggregation is the principal component of numerous protein misfolding pathologies termed proteinopathies, such as Alzheimer’s disease, Parkinson’s disease, prion disease, and AA amyloidosis with unmet treatment needs. Protein aggregation inhibitors have great potential for the prevention and treatment of proteinopathies. Here we report the development of an automated real-time microliter-scale high throughput screening (MSHTS) system for amyloid aggregation inhibitors using quantum-dot nanoprobes. Screening 504 crude extracts and 134 low molecular weight aromatic compounds revealed the relationship of amyloid-β (Aβ) aggregation inhibitory activities of plant extracts using a plant-based classification. Within the eudicots, rosids, Geraniales and Myrtales showed higher activity. Screening low molecular weight aromatic compounds demonstrated that the structure of tropolone endows it with potential Aβ aggregation inhibitory activity. The activity of the most active tropolone derivative was higher than that of rosmarinic acid. MSHTS also identified three chaperone molecules as tau aggregation inhibitors. These results demonstrate that our automated MSHTS system is a novel and robust tool that can be adapted to a wide range of compounds and aggregation-prone polypeptides.

## Introduction

Proteinopathies, which include most neurodegenerative diseases, are characterized by the aggregation of misfolded proteins^1^. For example, in Alzheimer’s disease (AD), the peptide amyloid-β (Aβ) and the protein tau aggregate in the brain of patients^2–4^. Therefore, the aggregation inhibitors of proteins have great potential as key compounds in the prevention, treatment, and research of proteinopathies.

In 2016, aducanumab, a human monoclonal antibody that selectively targets aggregated Aβ, reduced brain Aβ in a dose- and time-dependent manner and this advance was accompanied by a slowing of clinical decline in patients with prodromal or mild AD^5^. This suggests that it is appropriate to target Aβ aggregation to prevent and treat AD. Since the aggregation of Aβ and tau in the brain starts several decades before the onset of AD, it is more advantageous to initiate treatment and to prevent the targeting of these amyloid proteins as soon as possible. Recently, Nakamura *et al*. demonstrated that plasma biomarkers could be used to predict brain Aβ burden^6^, facilitating the early detection and treatment of AD. On the other hand, aducanumab and low molecular weight organic compounds such as polyphenols^7,8^ have been reported as drugs targeting Aβ, but none have been certified as medicine. It is therefore important to pursue other lead compounds that affect the aggregation of Aβ. Although inhibitory activity against amyloid aggregation is generally measured by spectrophotometric assays using thioflavin-T^9,10^, direct imaging of the aggregation using electronic microscopy^11–13^, and atomic force microscopy^14,15^, these methods are unsuitable for high-throughput analysis of natural extracts containing various contaminants because of false positives^16^ and low operability for high-throughput analysis.

Recently, we reported a real-time imaging method of Aβ aggregation using the Qdot nanoprobe^17,18^, and developed a microliter-scale high-throughput screening (MSHTS) system of Aβ aggregation inhibitors using the imaging method^19^. The advantages of the MSHTS system are small sample volume and high-throughput with a 1536-well plate, though some challenges remain such as the ability to generalize this system so that it can be used by anyone and for any sample. Therefore, in this study, we developed an automated system that can evaluate the protein aggregation inhibitory activity of a large number of heterogeneous samples rapidly and accurately.

## Results and Discussion

### Automation of MSHTS system

**Figure 1:**
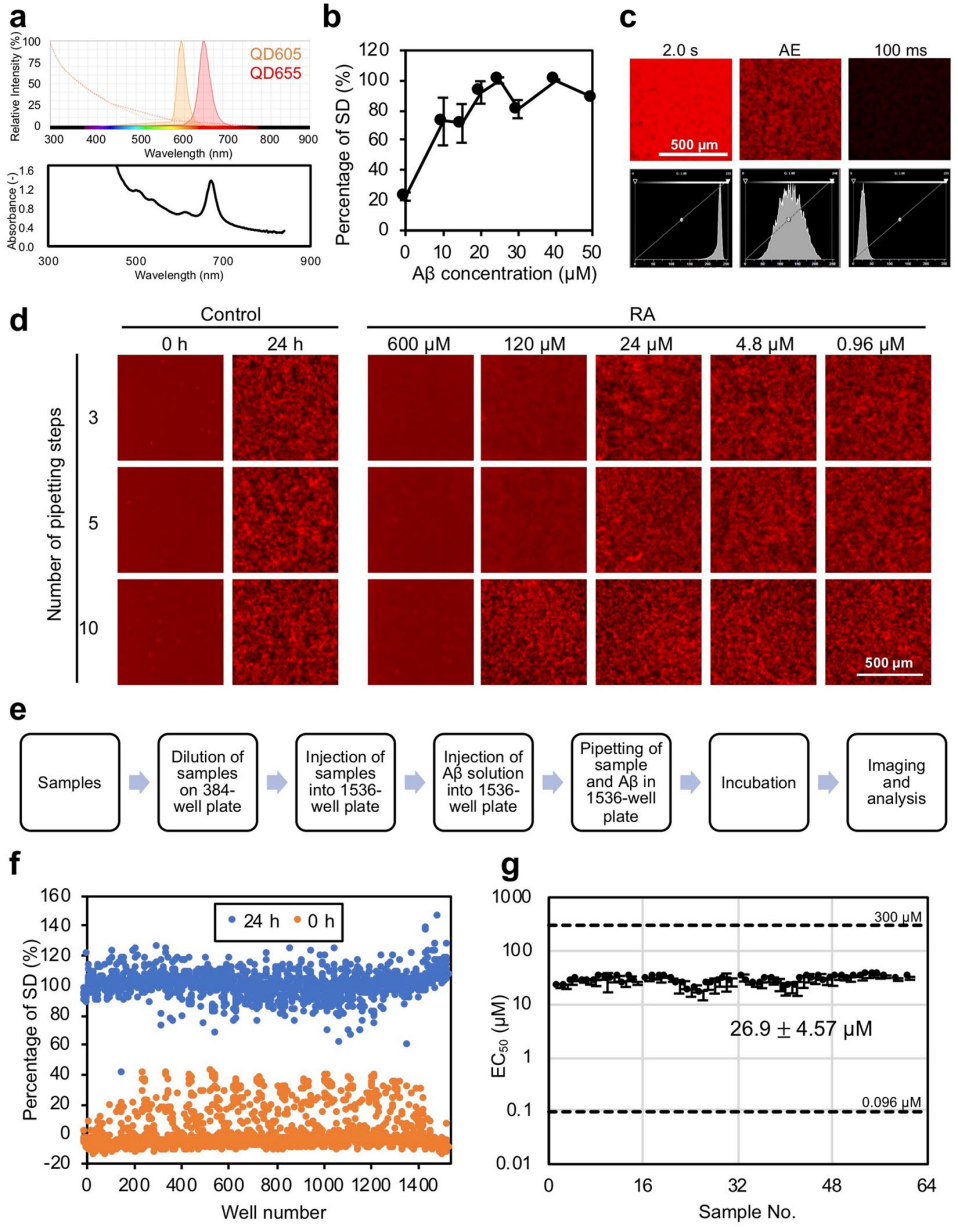
Development of the automated MSHTS system. (**a**) Fluorescence spectrums of QD 605/655 (top) and absorption spectrum of a typical plant (*Rosa rugosa*) extract. (**b**) Concentration-dependent Aβ aggregation. Various concentrations of Aβ_42_ and 30 nM QDAβ were incubated in a 1536-well plate at 37 °C for 24 h, then SD values were determined from fluorescence micrograph images. (**c**) Fluorescence micrographs acquired under various exposure conditions (top) and the histograms of fluorescence intensities of each pixel (bottom). Bright condition (left, exposure time = 2.0 s), optimum condition (middle, auto exposure), and dark condition (right, exposure time = 100 ms). (**d**) Relationship between number of pipetting steps and Aβ aggregation inhibitory activity by rosmarinic acid. (**e**) A scheme of the MSHTS system of Aβ aggregation inhibitors. (**f**,**g**) Validation of the automated MSHTS system. SD values of fluorescence images before (0 h) and after (24 h) incubation without inhibitors (**f**). EC_50_ values of rosmarinic acid determined using a 1536-well plate (Supplementary Fig. S6).

We initially optimized the previously reported MSHTS method^19^ before system automation. We used Qdot655 (Fig. 1a, top), which emits fluorescence at 655 nm, in previous reports^19–21^ on the MSHTS system. However, crude extracts of many plants have chlorophyll-derived absorption at 650–700 nm (Fig. 1a, bottom). Since this absorption may affect the quantification of amyloid aggregation inhibitory activity (Supplementary Fig. S1), we used Qdot605, which does not overlap with the absorption of chlorophylls (Fig. 1a), in this automated MSHTS system. We confirmed that the difference in Qdot type did not affect the measurement of aggregation inhibitory activity (Supplementary Fig. S2). In addition, we also confirmed blinking was not observed in both Qdot655 and Qdot605 nanoprobes under this condition using a 4x objective lens because blinking of a huge number of Qdot molecules in the field was averaged. Next, we examined the optimal Aβ concentration. In this system, we estimate the amount of Aβ aggregates from the variation in brightness of each pixel, as a standard deviation (SD) value according to a previous report^19^. Measurement of the Aβ concentration-dependent SD value (Fig. 1b) revealed that 25 µM Aβ showed the maximum SD value. As mentioned in a previous report^19^, SD values were correlated with the amount of Aβ aggregates when the thickness of the aggregate was within the range of the depth of focus. Therefore, we adopted 25 µM Aβ as the concentration at which the SD value showed a maximum value, because a wide range of SD values is directly related to the sensitivity of the system. Next, we examined the area in the center of the well to use quantification, and revealed that the size of the area did not significantly affect the SD value (Supplementary Fig. S3). In this study, we measured the SD value in the center region of 800 × 800 µm (432 × 432 pixels). In general, to calculate accurate SD values, histogram data needs to be normally distributed. In fact, pixel images that were too bright (Fig. 1c left) or too dark (Fig. 1c right) were not normally distributed. A detailed examination of the exposure time revealed a normal distribution in the histograms of the brightness of each pixel observed between 500 ms and 900 ms (Supplementary Fig. S4a). Furthermore, the SD value peaked during this period (Supplementary Fig. S4b). We further examined the camera gain to shorten the analysis time (Supplementary Fig. S5a). The SD values of Aβ aggregates did not significantly affect the tested range of camera gain (Supplementary Fig. S5b). However, since the SD values tended to rise slightly when camera gain was 32 or more (Supplementary Fig. S5b), we decided to set the camera gain at less than 32. Together with these results, we attempted to capture fluorescent images by increasing auto exposure to 160 ms and then camera gain to 32 (Supplementary Fig. S5c). At that time, the target maximum light intensity of the camera (Nikon, digital camera DS-Ri2) was set to 50%. All images were adjusted by a macro program so that average intensity became 50%. The SD values of each image were then determined using NIS elements. The SD value was also significantly affected by a defocused image. In this system, therefore, we used the XYZ Overview program of NIS elements or the Perfect Focus System (Nikon). With these settings, we were able to calculate SD values of all 1536 wells within 1 h.

To automate sample dilution and mixing with Aβ, in this study, we used JANUS G3 Automated workstation (PerkinElmer). The results demonstrate that the mixing process of Aβ and inhibitor significantly affected the evaluation of activity. When Aβ and rosmarinic acid (RA), which served as a positive control, were mixed by pipetting 10 times, 120 µM RA did not inhibit Aβ aggregation (Fig. 1d bottom). On the other hand, when the samples were mixed by pipetting three or five times, 120 µM RA inhibited Aβ aggregation (Fig. 1d top and middle). Generally, since agitation increases the collision frequency of protein molecules, it is well known that amyloid aggregation is promoted by agitation such as stirring and sonication. It is likely that the decrease of inhibitory activity by mixing (Fig. 1d bottom) is due to the aggregation-promoting effect by agitation being greater than the inhibition effect of RA. These results revealed an important implication related to mixing, namely that great care must be taken in the mixing step, by considering the type of pipetting tip, speed, volume, distance between tip and well bottom, etc., when evaluating amyloid aggregation inhibitory activity by spectrophotometric assays, other microscopic assays, and cytotoxicity assays.

An outline of the automated MSHTS system developed in this study is as follows (Fig. 1e). First, sample solutions, such as natural extracts and/or chemical compounds, were diluted stepwise in a 384-well plate. The diluted samples in this plate were injected into a 1536-well plate at 2.5 µl per well (Supplementary Fig. S6a). Next, 2.5 µl of 50 µM Aβ and 50 nM QD-labeled Aβ (Aβ solution) prepared in a cold 384-well plate at 4 °C were injected into a 1536-well plate containing diluted samples, and were mixed by pipetting three times. The 1536-well plate was sealed, and each well was imaged by a fluorescence microscope system. After incubation to induce aggregation for 24 h at 37 °C, each well was imaged by a fluorescence microscope system. The images were analyzed to determine the SD values of fluorescence intensity of each pixel. The half-maximal effective concentration (EC_50_) was estimated from the SD values according to our previous method^19^.

Finally, we verified the accuracy of the automated MSHTS system. All wells of the 1536-well plate that were incubated with 25 µM Aβ and 25 nM QDAβ were imaged before and after incubation by fluorescence microscopy, and SD values of fluorescence intensity of each pixel in these images were determined. The results show that the SD values of all wells increased significantly after 24 h of incubation (Fig. 1f). Figure 1g shows the positive control experiment using RA. When the concentration of RA was 300 and 60 µM, Aβ aggregation was inhibited (Supplementary Fig. S6b). The average EC_50_ of 61 sets was 26.9 ± 4.57 µM (Fig. 1g).

### Evaluation of Aβ aggregation inhibitory activity of methanolic extracts of 504 plants

**Figure 2:**
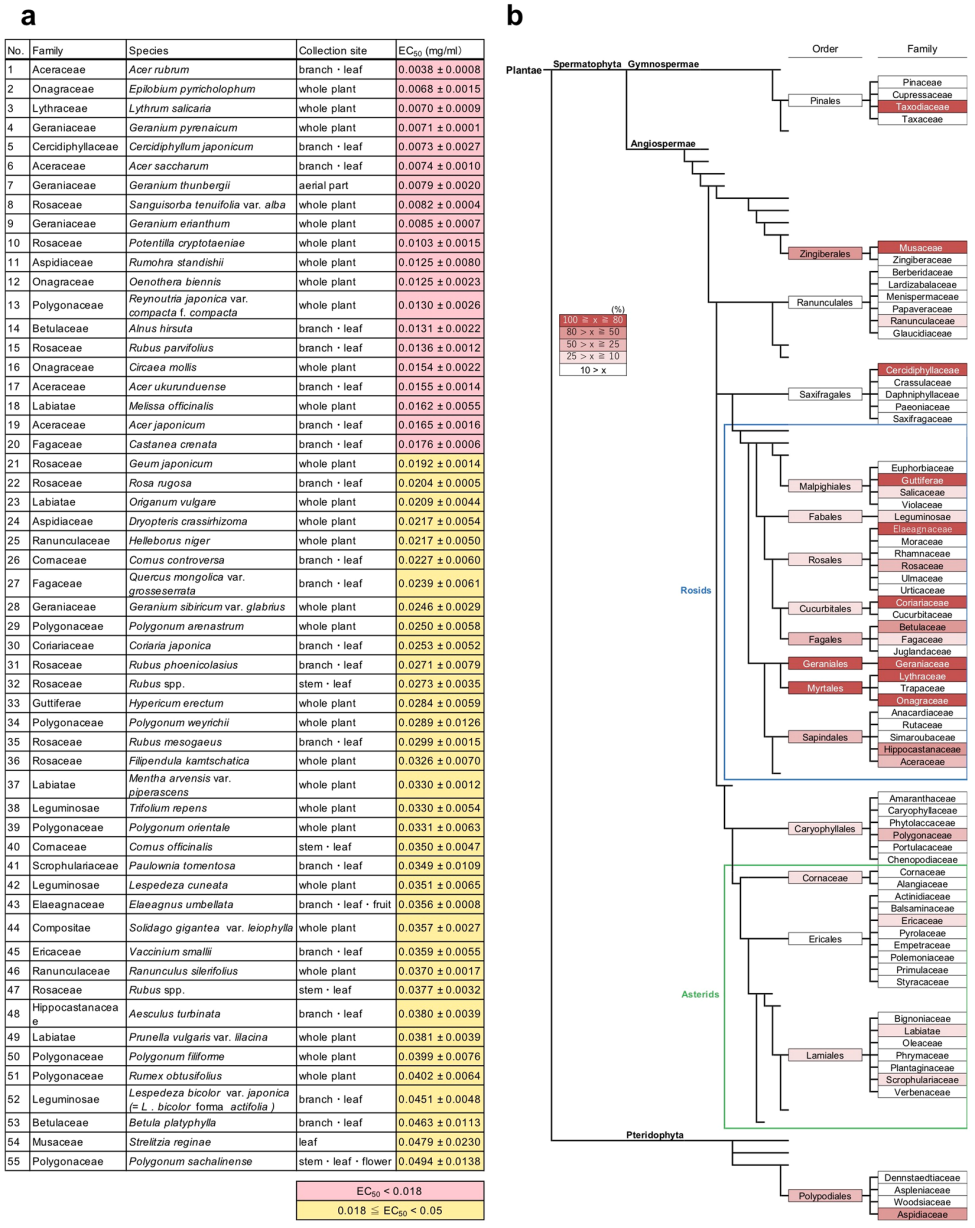
Ranking of 504 natural plant extracts for Aβ aggregation inhibitory activity. (**a**) Plant list showing EC_50_ values of 0.05 mg/ml or less (approximately top 10% activity). The number shows the rank of Aβ aggregation inhibitory activity. Activity with an EC_50_ value of 0.018 mg/ml (EC_50_ value of spearmint was the highest among the 52 spices^19^) or less is indicated in red while activity in the range of 0.018 to 0.05 mg/ml is indicated in yellow. (**b**) Order- and family-level analysis using the APG system. The percentage of highly active species included in each plant group is indicated as a red gradation.

The automated MSHTS system not only enables a microliter-scale and high-throughput analysis but is also able to evaluate crude extracts containing various substances, thereby showing an inner filter effect. Therefore, we evaluated the crude extracts from 504 plants collected in Hokkaido, Japan, using our automated MSHTS system. Since the plant extract library and the compound library that were evaluated in the following experiments were dissolved in DMSO, we confirmed whether DMSO affects Aβ aggregation (Supplementary Fig. S7a). The results show that the concentration of DMSO contamination during evaluation had no significant effect on Aβ aggregation (Supplementary Fig. S7b). In previous reports we revealed that approximately 90% of ethanol extracts of 52 spices and 11 seaweeds showed Aβ aggregation inhibitory activity in the presence of 10 mg/ml of sample^19,20^. To find plants with higher activity, samples were diluted to a concentration of 0.1 mg/ml or less, and their EC_50_ values were determined. The results of evaluation revealed that the EC_50_ values of 139 plant extracts were below 0.1 mg/ml, which was more active than rosemary extract (EC_50_ = 0.29 mg/ml)^19^ including RA (Supplementary Fig. S8, Supplementary Table S1). 55 plants showed high activity of 0.05 mg/ml or less, and 20 plants exhibited higher activity than spearmint (EC_50_ = 0.018 mg/ml), which was the most active spice^19^ (Fig. 2a). Interestingly, the Aβ aggregation inhibitory activities of plant extracts were related with plant classification. Among the 20 plants that were more active than spearmint^19^, 19 species were Eudicots (Supplementary Fig. S9). Among the Eudicots, the activity of rosids was high, and Geraniales and Myrtales were orders with particularly high activity (Fig. 2b). Geraniales with the highest activity were in the *Geranium* genus, including *G. sibiricum* var. *glabrius*, *G. pyrenaicum*, *G. erianthum*, and *G. thunbergii* (Supplementary Table S1), all belonging to the Geraniaceae. Geraniin is the main contributor of tannins in all investigated *Geranium* species^22^. Tannic acid dose-dependently inhibited Aβ aggregation^23^, suggesting that tannins can inhibit Aβ aggregation. We are now isolating the active compounds from plants that showed higher activity, and those results will be reported elsewhere in the near future.

### Screening a compound library by the MSHTS system

**Figure 3:**
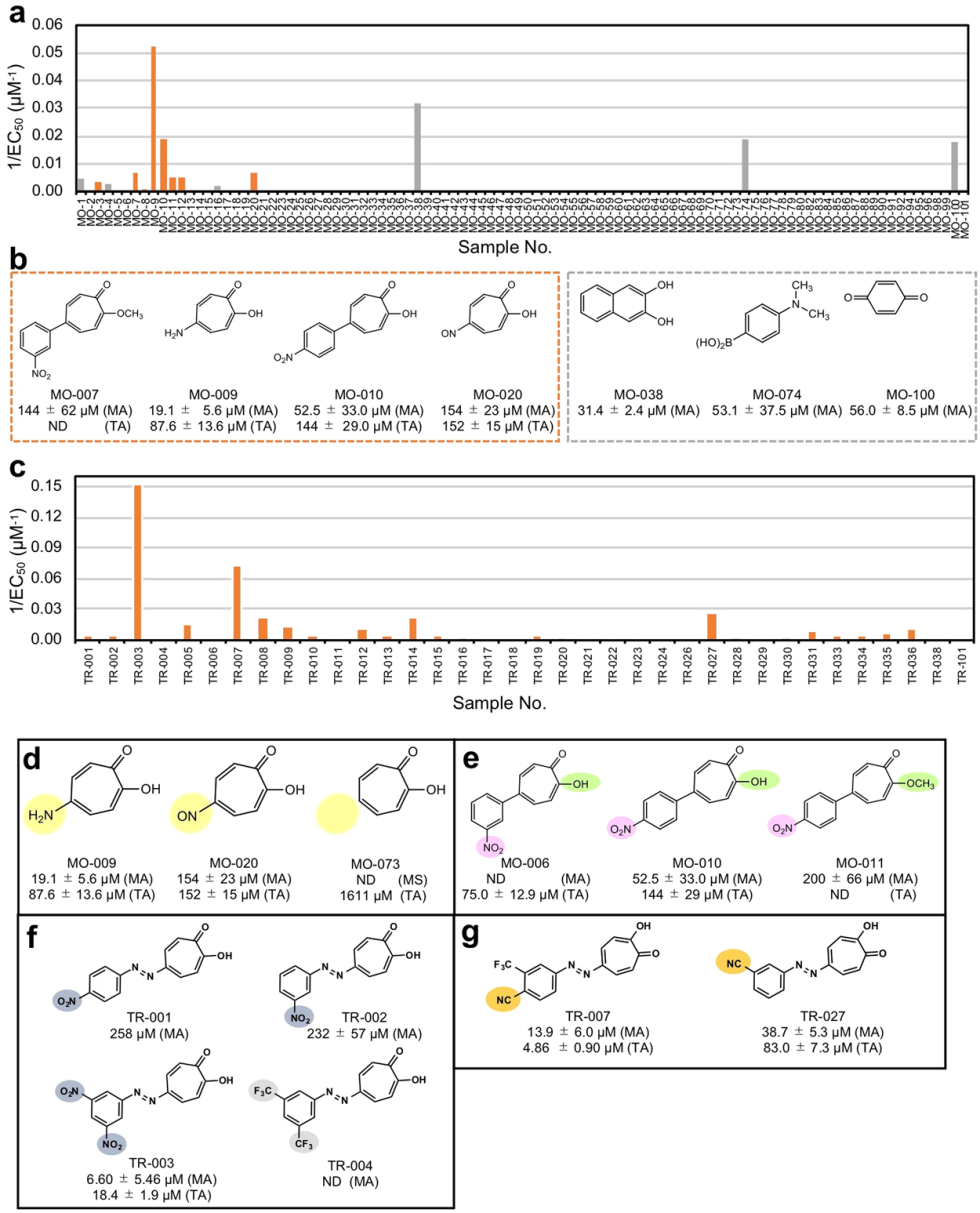
Screening of low molecular weight aromatic compounds. (**a**) Initial screening of the compound library. The horizontal axis represents sample number and the vertical axis represents the reciprocal EC_50_ values. Among the 98 tested samples, 13 samples showed Aβ aggregation inhibitory activity. Seven of these had a tropolone structure (orange bars). (**b**) Structure and activity of compounds showing EC_50_ values of 200 µM or less by the MSHTS system. (MA) and (TA) show EC_50_ values estimated by the MSHTS system and ThT assay, respectively. Compounds surrounded by a dotted orange line are compounds containing a tropolone structure. (**c**) Second screening of tropolone derivatives. (**d–g**) Comparison of Aβ aggregation inhibitory activity of tropolone derivatives with similar structures.

Next, we tried to screen aromatic low-molecular weight compounds by the MSHTS system to discover novel inhibitory compounds and important structures that are related to inhibition of Aβ aggregation (Fig. 3). We first screened 98 aromatic low-molecular weight compounds (Supplementary Table S2) using the MSHTS system, and consequently found 13 active compounds (MO-001, -003, -004, -007, -009, -010, -011, -012, -016, -020, -038, -074, and -100) (Fig. 3a). Interestingly, seven of the 13 compounds were tropolone derivatives (Fig. 3a, orange bars) and the inhibitory activity of MO-009 was higher than that of RA (Fig. 3b). Therefore, we prepared 36 novel tropolone derivatives (Supplementary Table S3), and determined their inhibitory activities. The results show that 25 derivatives had inhibitory activity (Fig. 3c, orange bars). TR-003 had the highest inhibitory activity (Fig. 3c). The tropolone derivatives that showed higher activity (EC_50_ < around 200 µM) were also subjected to a ThT assay (Supplementary Table S4), which confirmed that the majority of these compounds showed inhibitory activity (Fig. 3b,d–g). Differences between the MSHTS system and ThT assay may be due to differences in the detection mechanism, imaging of aggregates or interaction with the β sheet structure.

EC_50_ values obtained by MSHTS showed that functional groups strongly influenced inhibitory activity. The tropolone ring alone did not show inhibitory activity (Fig. 3d, right). The amino group (Fig. 3d, left) and nitric oxide group (Fig. 3d, middle) augmented inhibitory activity, suggesting that the electron status of other substituents on the tropolone ring affect inhibitory activity. When the EC_50_ values of MO-006 and MO-010 were compared, the position of the nitro group (*m*- or *p*-position) also appeared to affect inhibitory activity (Fig. 3e). The activity of MO-010 was higher than that of MO-011 whose hydroxyl group is masked by a methyl group. It is likely that the hydroxyl group of the tropolone ring is important for the inhibitory activity (Fig. 3e). A comparison between TR-001-004, and TR-003 showed the highest inhibitory activity (Fig. 3f). Since TR-001 and -002 had lower activity than TR-003, inhibitory activities might affect the number of electron-withdrawing units. TR-004 showed no activity, also suggesting that electron-withdrawing units as well as hydrophilicity contribute to inhibitory activity. Figure 3g suggests that the presence of a cyano group is involved in inhibitory activity. −NH_2_, −NO_2_, −OH, and −CN are functional groups that can form a hydrogen bond with Aβ_42_, suggesting that the number of hydrogen bonds and their steric position significantly affected Aβ aggregation inhibitory activity of tropolone derivatives.

### Extension of MSHTS system to screening tau aggregation inhibitors

In the brains of AD patients, hyperphosphorylated tau protein dissociates from the axonal microtubule and abnormally aggregates to form an insoluble paired helical filament (PHF). The formed PHF is deposited as neurofibrillary tangles (NFT). Since there is a correlation between frequency of occurrence of NFT and the progress of lesions, understanding the mechanism of tau aggregation and inhibition of this aggregation is important.

**Figure 4:**
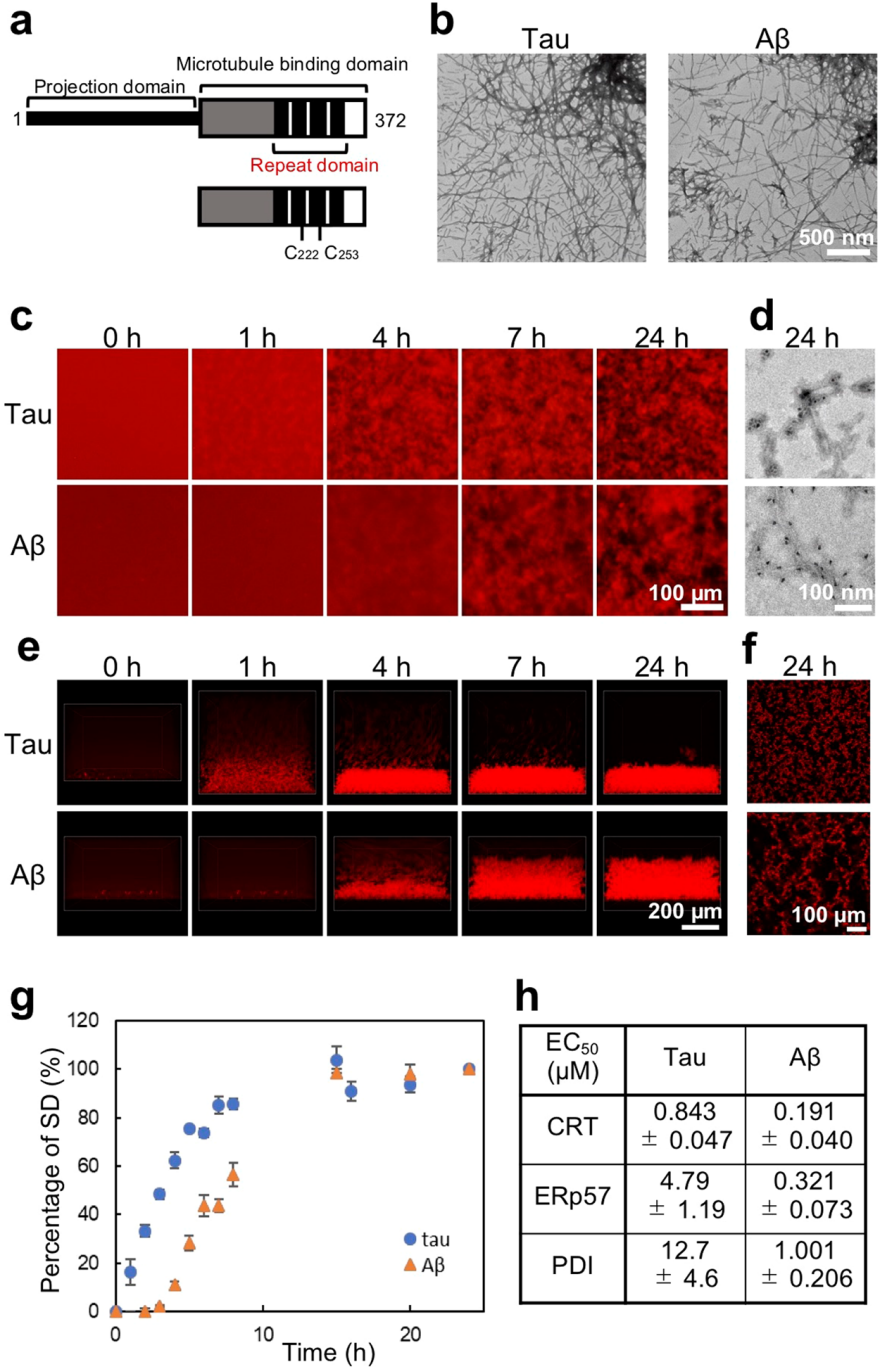
Imaging of tau aggregation and evaluation of aggregation inhibitory activity of chaperones. (**a**) Schematic structures of tau and tau MBD used in this study. (**b**) TEM images of tau and Aβ aggregates. (**c**) 2D imaging of tau and Aβ aggregation processes using QDTau and QDAβ. (**d**) TEM image of tau and Aβ aggregates with QD nanoprobes. (**e**) 3D time lapse imaging of tau and Aβ aggregation by confocal microscopy. (**f**) 2D images of tau and Aβ aggregates. (**g**) Percent of SD values determined by 2D image of (**c**). (**h**) Inhibitory activity of chaperone, CRT, ERp57, and PDI, for tau and Aβ aggregation (fluorescence images show Supplementary Fig. S12).

In this study, we used the microtubule-binding domain (MBD) fragment of 4-repeat tau (Fig. 4a) to image tau aggregation. First, we examined the aggregated condition of tau in the presence of heparin, which formed paired helical-like filaments under physiological conditions *in vitro*, according to early reports^24,25^. The results showed that the addition of DTT was necessary for aggregation of the MBD fragment of 4-repeat tau (Supplementary Fig. S10a,b). Since 4-repeat tau contains two Cys residues (C_222_ and C_253_), it may be necessary for aggregation to occur, to inhibit intramolecular disulfide crosslinking, as was reported in a previous report^26^. Since DTT also increased the affinity between tau and heparin but did not affect tau fibril morphology, and since normal neuronal cells were under a reducing environment^27^, we decided to add DTT in this assay. When 10 μM tau was incubated in the presence of 10 mM DTT, the SD value reached a maximum (Supplementary Fig. S10c,d), so we decided to observe aggregation using 10 μM tau. A sedimentation assay demonstrated that almost all tau fragments were recovered in the pellet (Supplementary Fig. S10d). We compared tau and Aβ aggregates by TEM observation. The morphology of tau and Aβ fibrils was difficult to distinguish (Fig. 4b). Real time imaging of tau and Aβ using QD showed that the aggregation of tau was faster than that of Aβ (Fig. 4d). We also confirmed by TEM observation that the aggregation of tau and Aβ contained QD nanoprobes (Fig. 4d). These aggregations were directly observed by confocal microscopy in real time (Fig. 4e). Interestingly, by using QD nanoprobes, we could distinguish aggregate shapes of tau and Aβ, which could not be identified by TEM observation (Fig. 4d). The imaging method using a QD nanoprobe without a drying step as in TEM observation may be suitable for detailed observation of aggregate shapes in solution. The SD values of the images demonstrated that no (or a short) lag time was observed in tau aggregation in this condition (Fig. 4g).

Since imaging of tau aggregation and quantification of the amount of aggregates from SD values were successful, we attempted to quantify the aggregation inhibitory activity of RA and spearmint extract that showed Aβ aggregation inhibitory activity^19^. The results revealed that the EC_50_ values of both RA and spearmint extracts could not be determined under these conditions (Supplementary Fig. S11), suggesting that the inhibitory activities of both extracts for tau aggregation were significantly lower than that for Aβ aggregation. We then tried to evaluate tau aggregation inhibition by using chaperones (calreticulin (CRT), ERp57, and Protein disulfide isomerase (PDI) (Supplementary Fig. S12) for which strong Aβ aggregation inhibitory activities have recently been reported^28^. The EC_50_ values suggested that CRT, ERp57, and PDI had inhibitory activity towards tau aggregation although their activities were lower than that for Aβ (Fig. 4h). Since tau aggregates accumulate in the cell, inducing the expression of chaperones may be an effective way to inhibit neurofibrillary tangle formation. The discovery of tau aggregation inhibitory substances localized in cells will help to understand the pathogenesis of AD and possibly prevent it.

### Future of the MSHTS system

In this study, we developed an automated MSHTS system using quantum-dot nanoprobes for Aβ aggregation inhibitors that can accurately and highly efficiently evaluate inhibitory activity even for crude extracts from natural products. The results of actual screening in this study and in our previous reports^19–21^ proved that this automated system is a powerful tool for discovering novel aggregation inhibitors and developing them as lead compounds.

Cadmium-based quantum dots used in this study are toxic to biological systems. The MSHTS system is an *in vitro* assay and there is no direct harm to the organism. However, since this system can use fluorescent probes other than Qdot, we would like to convert it into a safer fluorescent probe such as fluorescent carbon dots in the future.

Oligomerization inhibitors are more useful because recent studies have revealed that smaller oligomers of Aβ show higher neurotoxicity than fibrils^29,30^. The aggregation of Aβ proceeds in the order of oligomer, protofibrils, and fibril, suggesting that oligomerization and protofibrilization inhibitors also affect the amount of Aβ aggregates in the same incubation period. Therefore, although the MSHTS system quantifies only the fibrils of the final product of aggregation, it will be able to evaluate substances that inhibit the oligomer and protofibril formation steps in the course of aggregation. Whether the inhibitors found by the MSHTS system inhibit the oligomer formation step needs to be studied by another method. We are currently trying to develop an evaluation method of oligomerization inhibitory activity by applying real-time quantification of Aβ aggregation using the Qdot nanoprobe. By using this novel method and oligomer-specific antibodies, the inhibition stage of many inhibitors being found by the automated MSHTS system will be elucidated, and a list of more useful inhibitors, including the inhibitory stage, will be created. In this study, we showed that the mixing conditions significantly affect Aβ aggregation and its inhibition (Fig. 1d). This result implies that more careful examination of mixing conditions is also important in research on oligomer formation, which has attracted attention in recent years.

Although the structural diversity of inhibitors against a target molecule with high ligand specificity as enzymes is limited, various working points can be considered for aggregation inhibition of disordered proteins such as amyloid proteins. In other words, there are a wide variety of substances that function in various structural states such as oligomers, protofibrils, mature fibrils, and their structural polymorphisms. It is important to distinguish that screening for protein aggregation inhibitors is a different approach than finding the best way to develop traditional medicine. To find highly diverse substances that are effective against proteinopathies, a high-throughput evaluation system that can comprehensively evaluate various sources is extremely useful. We believe that this automated MSHTS system will be one of the most important analytical systems for screening protein aggregation inhibitors. The ability to analyze the inhibitor group using this screening system would allow for the development of therapeutic and preventive drugs, as well as functional foods, that could be used to prevent proteinopathies.

## Materials and Methods

### Materials

Human Aβ_42_ (4349-v, Peptide Institute) and Cys-conjugated Aβ_40_ (23519, Anaspec) were purchased commercially. Mouse tau MBD fragment was prepared as described in the Supplementary Methods. Chaperones, CRT, ERp57, and PDI, were prepared according to a previous report^31^. The plant extract library and the aromatic low-molecular weight compound library were prepared as described in the Supplementary Methods.

### Preparation of QDAβ and QDTau nanoprobes

QDAβ nanoprobe was prepared using QD-PEG-NH_2_ (Qdot^TM^ 655 ITK^TM^ Amino (PEG) Quantum dot; Q21521MP or Qdot^TM^ 605 ITK^TM^ Amino (PEG) Quantum dot; Q21501MP, Thermo Fisher Scientific) according to our previous report^19^. As for the QDAβ nanoprobe, QDTau was prepared as follows. 10 µM QD-PEG-NH_2_ was first reacted with 1 mM sulfo-EMCS (22307, Pierce) in PBS for 1 h at room temperature. After quenching and eliminating unreacted sulfo-EMCS, the QD-PEG-NH_2_-bound sulfo-EMCS was reacted with 62.5 µM of mouse tau MBD fragment for 1 h at room temperature. The concentrations of QDAβ and QDTau were determined by comparing absorbance at 350 nm to unlabeled QD-PEG-NH_2_.

### MSHTS systems

The automated MSHTS system used was based on the method described in the Supplementary Methods. In this study, EC_50_ values of the plant crude extract library and the compound library were measured by the automated MSHTS system and the manual MSHTS system^19,20^, respectively. All fluorescence micrograph images were taken using a 4x objective in the MSHTS systems.

### Fluorescence microscopy

Aggregates in the 1536-well plate were observed by an inverted fluorescence microscope (Nikon, TE2000) using a 4x objective equipped with a color CCD camera (DP72, Olympus).

### Confocal laser microscopy

Aggregates in the 1536-well plate were observed by a confocal laser microscope (Nikon, Nikon C2 Plus) using a 20x objective. Samples were excited at 561 nm by a TRITC laser. At each location, a z-stack with a step size of 4 µm (Aβ) and 2.5 µm (tau) was obtained with 512 × 512 pixels in each 2D image.

### TEM observations

Samples were deposited in 10 µl aliquots onto 200-mesh copper grids and negatively stained with 1% phosphotungstic acid. Specimens were examined under an H-7600 transmission electron microscope (Hitachi) at 60 kV.

### ThT assay

The ThT assay was conducted according to the method of Levine^10^ modified in our laboratory^19^.

## Supplementary information

Supplementary Information
