# Supplementary material for: An automated microliter-scale high-throughput screening system (MSHTS) for real-time monitoring of protein aggregation using quantum-dot nanoprobes
Source: Sci Rep. 2019 Feb 22;9:2587. doi: 10.1038/s41598-019-38958-0 (PMC6384891; doi:10.1038/s41598-019-38958-0)
Supplement: Supplementary file 1 — Supplementary Information [file 41598_2019_38958_MOESM1_ESM.pdf]

Supplementary information

An automated microliter-scale high-throughput screening system (MSHTS) for real-time monitoring of protein aggregation using quantum-dot nanoprobes

Rina Sasaki[1], Reina Tainaka[1], Yuichi Ando[1], Yurika Hashi[1,2], Hadya V. Deepak[3], Yoshiko Suga[3], Yuta Murai[3], Masaki Anetai[3], Kenji Monde[3], Kiminori Ohta[4,5], Ikuko Ito[6], Haruhisa Kikuchi[6], Yoshiteru Oshima[6], Yasuyuki Endo[4], Hitomi Nakao[7], Masafumi Sakono[7], Koji Uwai[1] & Kiyotaka Tokuraku[1]*

[1]Graduate School of Engineering, Muroran Institute of Technology, Muroran, Hokkaido, Japan. [2]Yamano College of Aesthetics, Hachioji, Tokyo, Japan. [3]Frontier Research Center for Advanced Material and Life Science, Faculty of Advanced Life Science, Hokkaido University, Sapporo, Hokkaido, Japan. [4]Faculty of Pharmaceutical Sciences, Tohoku Medical and Pharmaceutical University, Sendai, Miyagi, Japan. [5]School of Pharmacy, Showa University, Tokyo, Japan. [6]Graduate School of Pharmaceutical Sciences, Tohoku University, Sendai, Miyagi, Japan. [7]Graduate School of Science and Engineering, University of Toyama, Toyama, Toyama, Japan.

*Corresponding author: Kiyotaka Tokuraku Ph. D., Department of Applied Sciences, Muroran Institute of Technology, 27-1 Mizumoto-cho, Muroran 050-8585, Japan, Telephone: +81-143-46-5721, Fax: +81-143-46-5701, e-mail: tokuraku@mmm.muroran-it.ac.jp

This file includes:

Supplementary Methods

Supplementary Tables S1–4

Supplementary Figures S1–12

Supplementary Methods

<u>Automated MSHTS system–sample preparation and mixing with Aβ</u>

Sample preparation was carried out using Automated Workstation JANUS G3 (Perkin Elmer). To make a dilution series of samples, dilution buffer (10%EtOH, 1×PBS) was aspirated (asp. speed: 20 µl/s) using a conductive chip that can detect liquid level and injected (dsp. height: 0.75 mm, dsp. speed: 10 µl/s) into each well of a 384-well plate (384 Hard-shell micro plate HSP3951, BIO-RAD) without wells for injecting stock sample solution. Stock sample solutions were prepared, and were injected manually into the vacant wells of the 384-well plate. As a control without any inhibitor, dilution buffer alone was injected into the well instead of stock sample solution. The stock sample solution or control solution was aspirated (asp. height: 2 mm, asp. speed: 10 µl/s), injected (dsp. height: 4 mm, dsp. speed: 10 µl/s) into the next well containing the dilution buffer, and mixed by pipetting (asp. height: 2 mm, dsp. height: 4 mm, mix speed: 25 µl/s, mix cycles: 5). This dilution step was repeated five times, so that six dilution series were prepared for each sample. The diluted samples in the 384-well plate were aspirated using a 384-chip head (asp. height: 1 mm, asp. speed: 3 µl/s) and injected (quadruplicate) (dsp. height: 1 mm, dsp. speed: 10 µl/s) into a 1536-well plate (1536 well FIA black plate, Greiner Bio-One) at 2.5 µl per well. The Aβ solution, which is a mixture of 50 nM QDAβ and 50 µM Aβ42, was prepared in a microcentrifuge tube at 4 °C, aspirated (asp. speed: 30 µl/s) by a conductive chip that can detect liquid level, and injected (dsp. height: 0.5 mm, dsp. speed: 20 µl/s) into a cold 384-well plate placed at 4 °C. Finally, 2.5 µl of the Aβ solution were aspirated (asp. height: 1 mm, asp. speed: 3 µl/s) from the 384-well plate using a 384-chip head, injected (dsp. height: 0.5 mm, dsp. speed: 10 µl/s) into the 1536-well plate containing diluted samples, and mixed by pipetting three times (mix height: 0.5 mm, mix speed: 50 µl/s, mix volume: 3.5 µl, mix cycles: 3). The 1536-well plate was sealed with a plate seal (T-2417-8, BM Bio) to prevent evaporation. In order to remove bubbles and flatten the liquid surface, the 1536-well plate was centrifuged at 1,530 ×*g* for 5 min in a multi-well plate centrifuge (PlateSpin II, KUBOTA).

<u>Automated MSHTS system – Imaging and data analysis</u>

After centrifugation in a multi-well plate centrifuge, the 1536-well plate was observed by an inverted fluorescence microscope system (ECLIPSE Ti-E, Nikon) equipped with a color CCD camera (DS-Ri2, Nikon). QD was imaged using a 4× objective lens and a TRITC filter set (TRITC-A-Basic-NTE, Semrock). The images of each well were captured using an auto exposure up to 160 ms and then the camera gain was increased to 32. At that time, the target maximum light intensity of the camera setting was set to 50%, and the size of the region of interest (ROI) was set to 432 × 432 pixels at the center of each well. To focus on all wells of the 1536-well plate, we used the XYZ Overview program of NIS elements (Nikon) or the Perfect Focus System (Nikon). The 1536-well

plate was incubated at 37 °C for 1 day, and observed under the same conditions as indicated above. The SD value of the fluorescence intensity of each pixel was measured by the General Analysis program of NIS-Elements (Nikon) from ROI images (432 × 432 pixels). The half-maximal effective concentration (EC_{50}) was estimated from the SD values by Prism software (GraphPad) using an EC_{50} shift by global fitting (Asymmetric sigmoidal, 5 parameter logistic) according to our previous method[1].

Preparation of plant library

Five hundred and four plants growing in the wild or cultivated in Hokkaido were collected from June to November, 2004. Each was washed briefly, cut into small pieces using pruning shears and dried by hot-air at 50 °C for 24 h. Each dried material was powdered using a mixer before solvent extraction. Dried and powdered material (20 g for each sample) were extracted with 200 ml of 100% methanol at room temperature for 24 h. After filtration, the methanol solution was concentrated under reduced pressure to give a residue. Each crude extract was transferred to a 30 ml brown glass bottle and stored in a refrigerator at -20 °C. Average yields based on each plant's dry weight were about 25%. Each extract was dissolved in DMSO to a concentration of 100 mg/ml and the solution was stored in a refrigerator at -20 °C. A portion of each DMSO solution was used for several bioassays.

Preparation and management of drug solution of small molecule compound library

Each compound was dissolved in DMSO as a stock solution at a concentration of 20 mM, identified by a code number, and registered in the searchable database created by ChemBioFinder Ultra 12.0. Both 20 mM stock solutions and the pure compounds were stored at -80 °C in an ultra-cold freezer and used for bioassays after thawing with warm (35 °C) water.

Construction of plasmids for the bacterial expression of tau MBD fragment

The tau cDNAs were cloned from a mouse cDNA library. DNA sequencing analysis revealed that the sequences were identical to tau (Accession No.: NM_010838). To construct expression plasmids for tau MBD fragments, a primer set for the tau gene (tau-forward; 5′-AGACATATGAGCAGCCCCGGCTC-3′, tau-reverse; 5′-CCCAAGCTTTCACAAACCCTGCTTGGCC-3′), both of which contained cleavage sites for *Nde*I and *Hind*III, was purchased from Hokkaido System Science. Thirty rounds of amplification were carried out using the following parameters: denaturation at 98 °C for 10 s, annealing at 55 °C for 15 s, and primer extension at 72 °C for 60 s, on a TaKaRa PCR Thermal Cycler SP (Takara Bio). The purified PCR products and pET-21a (+) vector were digested with *Nde*I and *Hind*III, and purified using NucleoTrap (Macherey-Nagel) after agarose gel electrophoresis. The vector and insert

fragments were ligated using a DNA Ligation Kit (Takara Bio). The resultant plasmid expressed an N-terminal methionine residue, followed by a Pro-rich repeat, and tail regions of the genes for tau.

Preparation of tau MBD fragment

The bacterial expression and purification of tau MBD fragment was carried out as described previously[2]. Briefly, the expression plasmids were transformed into *E. coli* (Rosetta™ (DE3) pLys), and plasmid expression was induced by 1 mM isopropyl-1-thio-β-D-galactopyranoside. The heat-stable fraction of each extract was subjected to successive column chromatographies using a Bio-Scale™ Mini UNOsphere™ S (Bio-rad) and a TOYOPEARL® butyl column (Tosoh). Protein concentration was estimated using the method described by Lowry *et al.* (1951)[3], using bovine serum albumin (BSA) as the standard. Sodium dodecyl sulfate-polyacrylamide gel electrophoresis (SDS-PAGE) was carried out according to the method of Laemmli *et al.* (1970)[4].

Sedimentation assay of tau protein

10 μM tau, 50 nM QDTau, and 10 μM heparin in PBS were incubated in the presence or absence of 10 mM DTT in microtubes for 24 h at 37 °C and centrifuged at 386,000 ×*g* for 15 min at 4 °C. After removing the supernatant, the pellet was resuspended in the same volume of PBS. Supernatants and resuspended pellets were electrophoresed on an SDS-polyacrylamide gel according to the method of Laemmli (1970)[4].

Supplementary Table S1 List of EC_{50} values of 504 plant extracts determined by automated MSTHS system. Yellow and red cells indicate 0.018 μM ≤ EC_{50} < 0.05 μM and EC_{50} < 0.018 μM, respectively.

Family	Species	Collection site	EC_{50} (mg/ml)
Chloranthaceae	*Chloranthus japonicus*	whole plant	ND
Aristolochiaceae	*Asarum canadense*	branch・leaf	ND
Saururaceae	*Houttuynia cordata*	whole plant	ND
Lauraceae	*Cinnamomum zeylanicum*	branch・leaf	ND
Lauraceae	*Lindera umbellata* var. *membranacea*	stem・leaf	ND
Magnoliaceae	*Magnolia obovata*	branch・leaf	ND
Magnoliaceae	*Magnolia kobus* var. *borealis*	branch・leaf	ND
Magnoliaceae	*Magnolia acuminata*	branch・leaf・flower・fruit	ND
Araceae	*Epipremnum aureum*	vine・leaf	ND
Araceae	*Pinellia ternata*	whole plant	ND
Araceae	*Lysichiton camtschatcense*	whole plant	ND
Araceae	*Monstera deliciosa*	leaf	ND
Araceae	*Symplocarpus renifolius*	whole plant	ND
Araceae	*Arisaema serratum* (= *A. japonica*)	whole plant	ND
Dioscoreaceae	*Dioscorea batatas*	whole plant	ND
Liliaceae	*Liriope platyphylla*	whole plant	ND
Liliaceae	*Cardiocrinum cordatum* var. *glehnii*	whole plant	ND
Liliaceae	*Convallaria keiskei*	whole plant	ND
Liliaceae	*Allium schoenoprasum* var. *foliosum*	whole plant	ND
Liliaceae	*Polygonatum humile*	whole plant	ND
Liliaceae	*Convallaria majalis*	whole plant	ND
Liliaceae	*Smilax riparia* var. *ussuriensis*	whole plant	ND
Liliaceae	*Lilium lancifolium*	whole plant	ND
Liliaceae	*Hemerocallis fulva* var. *kwanso*	whole plant	ND
Liliaceae	*Smilacina japonica*	whole plant	ND
Liliaceae	*Polygonatum odoratum* var. *maximowiczii*	whole plant	ND
Liliaceae	*Allium tuberosum*	whole plant	ND
Liliaceae	*Disporum smilacinum*	whole plant	ND
Liliaceae	*Hosta rectifolia*	whole plant	ND
Liliaceae	*Ophiopogon japonicus*	whole plant	ND
Liliaceae	*Asparagus officinalis*	whole plant	ND
Liliaceae	*Disporum sessile*	whole plant	ND
Liliaceae	*Lilium medeoloides*	whole plant	ND
Liliaceae	*Veratrum grandiflorum* (= *V. album* var. *grandiflorum*)	whole plant	ND

Liliaceae	*Trillium kamtschaticum*	whole plant	ND
Liliaceae	*Polygonatum falcatum*	whole plant	ND
Liliaceae	*Paris tetraphylla*	whole plant	ND
Iridaceae	*Iris germanica*	whole plant	ND
Commelinaceae	*Commelina communis*	whole plant	0.0812
Cyperaceae	*Carex kobomugi*	whole plant	ND
Gramineae	*Miscanthus sinensis*	whole plant	ND
Gramineae	*Phragmites communis*	whole plant	ND
Gramineae	*Cymbopogon citratus*	leaf	ND
Gramineae	*Avena sativa*	whole plant	ND
Gramineae	*Setaria faberi* (= *S. autumnalis*; *S. macrocarpa*)	whole plant	ND
Gramineae	*Setaria glauca*	whole plant	ND
Gramineae	*Elymus mollis*	whole plant	ND
Gramineae	*Digitaria adscendens*	whole plant	ND
Gramineae	*Oplismenus undulatifolius*	whole plant	ND
Gramineae	*Zea mays*	whole plant	ND
Gramineae	*Coix lacryma-jobi* var. *mayuen*	branch・leaf	ND
Gramineae	*Sasa senanensis*	whole plant	ND
Typhaceae	*Typha latifolia*	stem・leaf	ND
Sparganiaceae	*Sparganium stoloniferum*	whole plant	ND
Musaceae	*Strelitzia reginae*	leaf	0.0479
Zingiberaceae	*Zingiber mioga*	whole plant	ND
Berberidaceae	*Berberis amurensis* var. *japonica*	branch・leaf	ND
Berberidaceae	*Berberis thunbergii*	branch・leaf	ND
Berberidaceae	*Epimedium diphyllum*	whole plant	ND
Berberidaceae	*Epimedium cremeum*	whole plant	ND
Berberidaceae	*Epimedium grandiflorum* subsp. *sempervirens* (= *E. sempervirens*)	whole plant	ND
Lardizabalaceae	*Akebia quinata*	vine・leaf	ND
Menispermaceae	*Cocculus laurifolius*	branch・leaf	ND
Papaveraceae	*Chelidonium majus* var. *asiaticum*	whole plant	ND
Papaveraceae	*Macleaya cordata*	whole plant	ND
Ranunculaceae	*Ranunculus silerifolius*	whole plant	0.0369
Ranunculaceae	*Thalictrum minus* var. *hypoleucum*	whole plant	ND
Ranunculaceae	*Clematis apiifolia*	vine・leaf・flower	ND
Ranunculaceae	*Helleborus niger*	whole plant	0.0217
Ranunculaceae	*Actaea asiatica*	whole plant	ND
Ranunculaceae	*Aconitum japonicum*	whole plant	ND
Ranunculaceae	*Clematis montana*	stem・leaf	ND
Ranunculaceae	*Ranunculus repens*	whole plant	ND

Ranunculaceae	*Clematis terniflora* (= *C. paniculata*)	branch・leaf	ND
Ranunculaceae	*Cimicifuga simplex*	whole plant	ND
Ranunculaceae	*Clematis ochotensis*	branch・leaf	ND
Ranunculaceae	*Nigella arvensis*	whole plant	ND
Ranunculaceae	*Coptis japonica*	whole plant	ND
Ranunculaceae	*Anemone hupehensis* var. *japonica*	whole plant	ND
Glaucidiaceae	*Glaucidium palmatum*	whole plant	ND
Platanaceae	*Platanus occidentalis*	branch・leaf	0.0562
Buxaceae	*Pachysandra terminalis*	whole plant	ND
Buxaceae	*Ilex crenata*	whole plant	ND
Cercidiphyllaceae	*Cercidiphyllum japonicum*	branch・leaf	0.0073
Crassulaceae	*Crassula portulacea*	branch・leaf	ND
Crassulaceae	*Sedum cauticolum*	whole plant	ND
Crassulaceae	*Sedum verticillatum*	whole plant	ND
Daphniphyllaceae	*Daphniphyllum macropodum* var. *humile*	branch・leaf	ND
Paeoniaceae	*Paeonia lactiflora*	whole plant	0.0731
Saxifragaceae	*Hydrangea macrophylla* var. *thunbergii*	branch・leaf	ND
Saxifragaceae	*Saxifraga sachalinensis*	whole plant	0.0765
Saxifragaceae	*Saxifraga fortunei* var. *incisolobata*	whole plant	0.0866
Saxifragaceae	*Hydrangea petiolaris*	branch・leaf	ND
Saxifragaceae	*Schizophragma hydrangeoides*	branch・leaf	ND
Saxifragaceae	*Hydrangea paniculata*	branch・leaf	ND
Saxifragaceae	*Astilbe thunbergii* var. *congesta*	whole plant	0.0721
Saxifragaceae	*Saxifraga stolonifera*	whole plant	0.0877
Saxifragaceae	*Hydrangea macrophylla*	stem・leaf	ND
Saxifragaceae	*Ribes japonicum*	branch・leaf	0.0740
Saxifragaceae	*Saxifraga japonica*	whole plant	ND
Saxifragaceae	*Chrysosplenium grayanum*	whole plant	ND
Loranthaceae	*Viscum album* var. *coloratum*	stem・leaf	ND
Amaranthaceae	*Achyranthes fauriei*	whole plant	ND
Amaranthaceae	*Amaranthus retroflexus*	whole plant	ND
Caryophyllaceae	*Silene dioica*	whole plant	ND
Caryophyllaceae	*Saponaria officinalis*	whole plant	ND
Caryophyllaceae	*Stellaria aquatica*	whole plant	ND
Caryophyllaceae	*Dianthus superbus* var. *longicalycinus*	whole plant	ND
Caryophyllaceae	*Stellaria media*	whole plant	ND
Phytolaccaceae	*Phytolacca americana*	stem・leaf	ND
Phytolaccaceae	*Phytolacca esculenta*	whole plant	ND
Polygonaceae	*Polygonum filiforme*	whole plant	0.0399

Polygonaceae	*Polygonum arenastrum*	whole plant	0.0250
Polygonaceae	*Polygonum nepalense*	whole plant	0.0646
Polygonaceae	*Rumex acetosa*	whole plant	ND
Polygonaceae	*Reynoutria japonica* var. *compacta* f. *compacta*	whole plant	0.0130
Polygonaceae	*Polygonum sachalinense*	stem・leaf・flower	0.0494
Polygonaceae	*Persicaria lapathifolia*	whole plant	0.0532
Polygonaceae	*Polygonum hydropiper*	whole plant	0.0606
Polygonaceae	*Polygonum orientale*	whole plant	0.0331
Polygonaceae	*Polygonum dumetorum*	vine・leaf・fruit	ND
Polygonaceae	*Polygonum weyrichii*	whole plant	0.0289
Polygonaceae	*Polygonum longisetum*	whole plant	ND
Polygonaceae	*Polygonum thunbergii*	whole plant	ND
Polygonaceae	*Polygonum cuspidatum*	whole plant	ND
Polygonaceae	*Persicaria sieboldii*	whole plant	ND
Polygonaceae	*Fagopyrum esculentum*	whole plant	ND
Polygonaceae	*Polygonum perfoliatum*	aerial part	ND
Polygonaceae	*Rumex obtusifolius*	whole plant	0.0402
Portulacaceae	*Portulaca oleracea*	whole plant	ND
Chenopodiaceae	*Chenopodium album* var. *centrorubrum*	whole plant	ND
Chenopodiaceae	*Chenopodium album*	whole plant	ND
Chenopodiaceae	*Salsola komarovii*	whole plant	ND
Vitaceae	*Parthenocissus tricuspidata*	vine・leaf	0.0832
Vitaceae	*Vitis coignetiae*	branch・leaf	ND
Vitaceae	*Parthenocissus quinquefolia*	vine	ND
Vitaceae	*Parthenocissus quinquefolia*	leaf	ND
Vitaceae	*Cayratia japonica*	vine・leaf	ND
Celastraceae	*Euonymus sieboldianus*	branch・leaf	0.0548
Celastraceae	*Euonymus oxyphyllus*	branch・leaf	ND
Celastraceae	*Euony fortunei*	branch・leaf	ND
Celastraceae	*Euonymus alatus* forma *striatus*	branch・leaf	ND
Celastraceae	*Euonymus alatus*	branch・leaf	ND
Oxalidaceae	*Oxalis corniculata*	whole plant	ND
Euphorbiaceae	*Euphorbia pulcherrima*	branch・leaf	0.0716
Guttiferae	*Hypericum erectum*	whole plant	0.0284
Salicaceae	*Salix bakko*	branch・leaf	0.0633
Salicaceae	*Populus alba*	branch・leaf	ND
Salicaceae	*Populus sieboldii*	branch・leaf	ND
Salicaceae	*Populus euroamericana*	branch・leaf	ND
Salicaceae	*Salix sachalinensis*	branch・leaf	ND

Salicaceae	*Salix integra*	branch・leaf	ND
Salicaceae	*Salix miyabeana*	branch・leaf	ND
Violaceae	*Viola kusanoana*	whole plant	0.0600
Coriariaceae	*Coriaria japonica*	branch・leaf	0.0253
Cucurbitaceae	*Gynostemma pentaphyllum*	aerial part	ND
Cucurbitaceae	*Thladiantha dubia*	branch・leaf	ND
Cucurbitaceae	*Schizopepon bryoniaefolius*	aerial part	ND
Cucurbitaceae	*Actinostemma lobatum*	whole plant	ND
Leguminosae	*Lathyrus japonicus*	whole plant	ND
Leguminosae	*Lupinus luteus*	branch・leaf	ND
Leguminosae	*Albizia julibrissin*	branch・leaf	ND
Leguminosae	*Amphicarpaea edgeworthii* var. *japonica*	whole plant	ND
Leguminosae	*Pueraria lobata*	vine・leaf	ND
Leguminosae	*Vicia cracca*	aerial part・flower	ND
Leguminosae	*Wisteria floribunda*	branch・leaf	0.0563
Leguminosae	*Trifolium pratense*	whole plant	ND
Leguminosae	*Melilotus suaveolens*	whole plant・flower	ND
Leguminosae	*Robinia pseudo-acacia*	branch・leaf	ND
Leguminosae	*Glycine max*	whole plant	ND
Leguminosae	*Gleditsia japonica*	branch・leaf	ND
Leguminosae	*Melilotus alba*	whole plant・flower	ND
Leguminosae	*Lespedeza bicolor* var. *japonica* (= *L. bicolor* forma *actifolia*)	branch・leaf	0.0451
Leguminosae	*Trifolium repens*	whole plant	0.0330
Leguminosae	*Vicia japonica*	aerial part	ND
Leguminosae	*Maackia amurensis* var. *buergeri*	branch・leaf	ND
Leguminosae	*Arachis hypogaea*	whole plant	ND
Leguminosae	*Medicago sativa*	whole plant	ND
Leguminosae	*Lespedeza bicolor*	branch・leaf	0.0537
Leguminosae	*Astragalus membranaceus* var. *mongholicus* (= *A. mongholicus*)	whole plant	ND
Leguminosae	*Sophora flavescens*	branch・leaf・fruit	ND
Leguminosae	*Astragalus membranaceus*	whole plant	ND
Leguminosae	*Desmodium fallax* var. *mandshuricum*	whole plant	0.0938
Leguminosae	*Cytisus scoparius*	branch・leaf	ND
Leguminosae	*Lespedeza cuneata*	whole plant	0.0351
Leguminosae	*Trifolium campestre*	whole plant	ND
Leguminosae	*Lotus corniculatus* var. *corniculatus*	whole plant	ND
Betulaceae	*Alnus hirsuta*	branch・leaf	0.0131
Betulaceae	*Ostrya japonica*	branch・leaf	ND
Betulaceae	*Alnus japonica*	branch・leaf	0.0620

Betulaceae	*Betula platyphylla*	branch・leaf	0.0463
Fagaceae	*Quercus dentata*	branch・leaf	0.0733
Fagaceae	*Quercus mongolica* var. *grosseserrata*	branch・leaf	0.0239
Fagaceae	*Castanea crenata*	branch・leaf	0.0176
Fagaceae	*Quercus rubra*	branch・leaf	0.0954
Fagaceae	*Fagus crenata*	branch・leaf	0.0976
Juglandaceae	*Juglans ailanthifolia*	branch・leaf・fruit	0.0724
Juglandaceae	*Juglans regia* var. *orientis*	branch・leaf	0.0696
Elaeagnaceae	*Elaeagnus umbellata*	branch・leaf・fruit	0.0356
Moraceae	*Ficus elastica*	branch・leaf	ND
Moraceae	*Morus bombycis*	branch・leaf	ND
Moraceae	*Humulus lupulus*	vine・leaf・fruit	ND
Moraceae	*Humulus scandens* (= *H. japonicus*)	vine・leaf	0.0916
Rhamnaceae	*Rhamnus japonica* var. *decipiens* (= *R. japonica*)	branch・leaf	ND
Rosaceae	*Rubus* spp.	stem・leaf	0.0273
Rosaceae	*Potentilla cryptotaeniae*	whole plant	0.0104
Rosaceae	*Rosa rugosa*	branch・leaf	0.0204
Rosaceae	*Sanguisorba tenuifolia* var. *alba*	whole plant	0.0082
Rosaceae	*Rubus mesogaeus*	branch・leaf	0.0299
Rosaceae	*Sorbus alnifolia*	branch・leaf	ND
Rosaceae	*Rosa setigera*	stem・leaf	0.0857
Rosaceae	*Duchesnea chrysantha*	whole plant	0.0582
Rosaceae	*Prunus padus*	branch・leaf	ND
Rosaceae	*Rubus parvifolius*	branch・leaf	0.0136
Rosaceae	*Spiraea thunbergii*	branch・leaf	0.0681
Rosaceae	*Rubus* spp.	stem・leaf	0.0377
Rosaceae	*Geum japonicum*	whole plant	0.0192
Rosaceae	*Agrimonia pilosa*	whole plant	0.0870
Rosaceae	*Prunus avium*	branch・leaf	ND
Rosaceae	*Aruncus dioicus* var. *tenuifolius*	whole plant	0.0642
Rosaceae	*Pyrus pyrifolia* var. *culta*	branch・leaf	ND
Rosaceae	*Rubus phoenicolasius*	branch・leaf	0.0271
Rosaceae	*Rosa multiflora*	branch・leaf	0.0870
Rosaceae	*Cotoneaster salicifolius*	branch・leaf	0.0562
Rosaceae	*Filipendula kamtschatica*	whole plant	0.0326
Rosaceae	*Prunus persica*	branch・leaf	ND
Rosaceae	*Sorbus commixta*	branch・leaf	ND
Rosaceae	*Aronia melanocarpa*	leaf	ND
Rosaceae	*Prunus ssiori*	branch・leaf	ND

Rosaceae	*Malus halliana*	branch・leaf	ND
Rosaceae	*Prunus mume* var. *bungo*	branch・leaf	ND
Rosaceae	*Cotoneaster horizontalis*	branch・leaf	0.0539
Rosaceae	*Eriobotrya japonica*	branch・leaf	ND
Rosaceae	*Malus baccata* var. *mandshurica*	branch・leaf	ND
Rosaceae	*Kerria japonica*	branch・leaf	0.0853
Rosaceae	*Spiraea cantoniensis*	branch・leaf	0.0849
Rosaceae	*Chaenomeles speciosa*	branch・leaf	ND
Rosaceae	*Prunus mume*	branch・leaf	ND
Rosaceae	*Malus sieboldii*	branch・leaf	ND
Rosaceae	*Prunus tomentosa*	branch・leaf	ND
Ulmaceae	*Zelkova serrata*	branch・leaf	ND
Ulmaceae	*Ulmus japonica*	branch・leaf	0.0873
Ulmaceae	*Ulmus laciniata*	branch・leaf	0.0759
Urticaceae	*Pilea mongolica*	whole plant	ND
Urticaceae	*Laportea bulbifera*	whole plant	ND
Urticaceae	*Boehmeria tricuspis*	whole plant	ND
Urticaceae	*Urtica platyphylla*	whole plant	ND
Geraniaceae	*Geranium sibiricum* var. *glabrius*	whole plant	0.0246
Geraniaceae	*Geranium pyrenaicum*	whole plant	0.0071
Geraniaceae	*Geranium erianthum*	whole plant	0.0085
Geraniaceae	*Geranium thunbergii*	aerial part	0.0079
Lythraceae	*Lythrum salicaria*	whole plant	0.0070
Trapaceae	*Trapa japonica*	stem・leaf	0.0858
Onagraceae	*Oenothera biennis*	whole plant	0.0125
Onagraceae	*Epilobium pyrricholophum*	whole plant	0.0068
Onagraceae	*Circaea mollis*	whole plant	0.0154
Cruciferae	*Sisymbrium officinale*	whole plant	ND
Cruciferae	*Raphanus raphanistrum*	whole plant	ND
Cruciferae	*Isatis tinctoria* var. *yezoensis*	aerial part・seed	ND
Cruciferae	*Rorippa sylvestris*	whole plant	0.0810
Cruciferae	*Cochlearia armoracia* (= *Armoracia rusticana*)	whole plant	ND
Cruciferae	*Cardamine flexuosa*	whole plant	ND
Cruciferae	*Arabis* (*stelleri* var.) *japonica*	whole plant	ND
Cruciferae	*Nasturtium officinale*	whole plant	ND
Cruciferae	*Cardamine leucantha*	whole plant	ND
Malvaceae	*Malva moschata*	whole plant	0.0665
Malvaceae	*Hibiscus syriacus*	branch・leaf	ND
Malvaceae	*Malva neglecta*	whole plant	ND

Sterculiaceae	*Sterculia nobilis*	branch・leaf	ND
Tiliaceae	*Corchorus olitorius*	branch・leaf	ND
Tiliaceae	*Tilia japonica*	branch・leaf	ND
Tiliaceae	*Tilia maximowicziana*	branch・leaf	ND
Thymelaeaceae	*Daphne kamtschatica* var. *jezoensis*	branch・leaf	0.0672
Anacardiaceae	*Rhus javanica*	branch・leaf	ND
Rutaceae	*Phellodendron amurense*	branch・leaf	ND
Rutaceae	*Skimmia japonica*	whole plant	ND
Rutaceae	*Zanthoxylum piperitum*	branch・leaf	0.0859
Simaroubaceae	*Ailanthus altissima*	branch・leaf	0.0630
Hippocastanaceae	*Aesculus glabra*	branch・leaf	ND
Hippocastanaceae	*Aesculus turbinata*	branch・leaf	0.0380
Aceraceae	*Acer saccharum*	branch・leaf	0.0074
Aceraceae	*Acer rubrum*	branch・leaf	0.0038
Aceraceae	*Acer japonicum*	branch・leaf	0.0165
Aceraceae	*Acer miyabei*	branch・leaf	0.0562
Aceraceae	*Acer palmatum* (var. *matsumurae*)	branch・leaf	ND
Aceraceae	*Acer negundo*	branch・leaf	ND
Aceraceae	*Acer ukurunduense*	branch・leaf	0.0155
Aceraceae	*Acer mono* var. *mayrii*	branch・leaf	ND
Aceraceae	*Acer mono*	branch・leaf	0.0887
Cornaceae	*Cornus controversa*	branch・leaf	0.0227
Cornaceae	*Cornus officinalis*	stem・leaf	0.0349
Alangiaceae	*Alangium platanifolium* var. *trilobum*	branch・leaf	ND
Actinidiaceae	*Actinidia chinensis*	vine・leaf	ND
Actinidiaceae	*Actinidia polygama*	branch・leaf	ND
Actinidiaceae	*Actinidia arguta*	branch・leaf	ND
Balsaminaceae	*Impatiens balfourii*	whole plant	ND
Balsaminaceae	*Impatiens noli-tangere*	whole plant	ND
Ericaceae	*Rhododendron dauricum*	branch・leaf	0.0599
Ericaceae	*Vaccinium hirtum*	branch・leaf	0.0888
Ericaceae	*Vaccinium ovalifolium*	branch・leaf	0.0782
Ericaceae	*Rhododendron schlippenbachii*	branch・leaf	0.0738
Ericaceae	*Enkianthus campanulatus*	branch・leaf	ND
Ericaceae	*Vaccinium japonicum* (= *Hugeria japonica*)	branch・leaf	ND
Ericaceae	*Rhododendron brachycarpum*	branch・leaf	ND
Ericaceae	*Rhododendron mucronatum*	branch・leaf	0.0568
Ericaceae	*Vaccinium smallii*	branch・leaf	0.0359
Ericaceae	*Erica* sp.	branch・leaf	ND

Pyrolaceae	*Pyrola incarnata*	aerial part	0.0605
Pyrolaceae	*Pyrola renifolia*	aerial part	ND
Pyrolaceae	*Chimaphila japonica*	whole plant	ND
Empetraceae	*Empetrum nigrum* var. *japonicum*	branch・leaf	ND
Polemoniaceae	*Polemonium yezoense*	whole plant	ND
Polemoniaceae	*Phlox drummondii*	whole plant	ND
Primulaceae	*Lysimachia vulgaris* var. *davurica*	whole plant	ND
Styracaceae	*Styrax obassia*	branch・leaf	ND
Boraginaceae	*Symphytum officinale*	whole plant	ND
Boraginaceae	*Borago officinalis*	whole plant	0.0653
Boraginaceae	*Lithospermum erythrorhizon*	whole plant	0.0835
Apocynaceae	*Vinca major*	whole plant	ND
Asclepiadaceae	*Metaplexis japonica*	whole plant	ND
Asclepiadaceae	*Cynanchum caudatum*	whole plant	0.0934
Asclepiadaceae	*Gomphocarpus fruticosus*	stem・leaf	ND
Asclepiadaceae	*Cynanchum sublanceolatum* var. *macranthum*	stem・leaf	ND
Gentianaceae	*Tripterospermum japonicum*	whole plant	ND
Gentianaceae	*Gentiana lutea*	whole plant	ND
Rubiaceae	*Coffea arabica*	branch・leaf	ND
Rubiaceae	*Asperula odorata*	whole plant	ND
Bignoniaceae	*Catalpa ovata*	branch・leaf	0.0733
Bignoniaceae	*Jacaranda filicifolia*	branch・leaf	ND
Labiatae	*Nepeta cataria*	whole plant	ND
Labiatae	*Origanum vulgare*	whole plant	0.0209
Labiatae	*Elsholtzia ciliata*	whole plant	ND
Labiatae	*Salvia officinalis*	stem・leaf	Nd
Labiatae	*Melissa officinalis*	whole plant	0.0162
Labiatae	*Stachys lanata*	stem・leaf	ND
Labiatae	*Leonurus sibiricus*	whole plant	0.0768
Labiatae	*Lamium barbatum*	whole plant	ND
Labiatae	*Perilla frutescens*	whole plant	0.0745
Labiatae	*Prunella vulgaris* var. *lilacina*	whole plant	0.0381
Labiatae	*Mentha arvensis* var. *piperascens*	whole plant	0.0330
Labiatae	*Tiarella polyphylla*	whole plant	0.0954
Labiatae	*Clinopodium gracile* var. *sachalinense*	whole plant	ND
Labiatae	*Stachys japonica* var. *villosa*	whole plant	ND
Labiatae	*Glechoma hederacea* var. *grandis*	whole plant	ND
Labiatae	*Scutellaria baicalensis*	whole plant	0.0697
Labiatae	*Lavandula vera*	whole plant	0.0696

Labiatae	*Salvia guaranitica*	whole plant	ND
Labiatae	*Perilla frutescens* var. *acuta* forma *viridis*	whole plant	ND
Labiatae	*Lycopus uniflorus*	whole plant	0.0501
Oleaceae	*Fraxinus mandshurica* var. *japonica*	branch・leaf	0.0605
Oleaceae	*Ligustrum tschonoskii*	branch・leaf	ND
Oleaceae	*Syringa reticulata*	branch・leaf	ND
Oleaceae	*Forsythia suspensa*	branch・leaf	ND
Oleaceae	*Forsythia viridissima*	branch・leaf	ND
Oleaceae	*Syringa vulgaris*	branch・leaf	ND
Oleaceae	*Ligustrum obtusifolium*	branch・leaf	ND
Phrymaceae	*Phryma leptostachya* var. *asiatica*	whole plant	ND
Plantaginaceae	*Plantago asiatica*	whole plant	0.0651
Plantaginaceae	*Plantago lanceolata*	whole plant	0.0945
Scrophulariaceae	*Paulownia tomentosa*	branch・leaf	0.0349
Scrophulariaceae	*Verbascum blattaria*	whole plant	ND
Scrophulariaceae	*Verbascum thapsus*	whole plant	ND
Scrophulariaceae	*Mazus miquelii*	whole plant	ND
Scrophulariaceae	*Antirrhinum majus*	whole plant	ND
Scrophulariaceae	*Linaria vulgaris*	whole plant	ND
Verbenaceae	*Callicarpa japonica*	branch・leaf	0.0792
Verbenaceae	*Clerodendron trichotomum*	branch・leaf	ND
Convolvulaceae	*Calystegia japonica*	whole plant	ND
Convolvulaceae	*Calystegia soldanella*	whole plant	ND
Solanaceae	*Physalis angulata*	whole plant	ND
Solanaceae	*Datura stramonium*	whole plant	ND
Solanaceae	*Lycium chinense*	branch・leaf・fruit	ND
Solanaceae	*Datura innoxia* (= *D. meteloides*)	stem・leaf	0.0654
Solanaceae	*Solanum nigrum*	whole plant	ND
Solanaceae	*Atropa belladonna*	whole plant	ND
Solanaceae	*Solanum japonense*	whole plant	ND
Solanaceae	*Solanum sarachoides*	whole plant	ND
Solanaceae	*Physalis alkekengi* var. *franchetii*	whole plant	ND
Aquifoliaceae	*Ilex crenata* var. *paludosa*	branch・leaf	ND
Compositae	*Calendula officinalis*	whole plant	ND
Compositae	*Aster novi-belgii*	whole plant	ND
Compositae	*Artemisia princeps* (= *A. vulgaris* var. *indica*)	whole plant	ND
Compositae	*Erigeron annuus*	whole plant	ND
Compositae	*Anthemis nobilis*	whole plant	ND
Compositae	*Anaphalis margaritacea* var. *angustior*	whole plant	ND

Compositae	*Solidago virgaurea* subsp. *asiatica*	whole plant	ND
Compositae	*Cacalia auriculata* var. *kamtschatica*	whole plant	ND
Compositae	*Aster novae-angliae*	whole plant	ND
Compositae	*Solidago gigantea* var. *leiophylla*	whole plant	0.0357
Compositae	*Artemisia montana*	whole plant	ND
Compositae	*Artemisia* (*japonica* subsp.) *littoricola*	whole plant	ND
Compositae	*Erigeron canadensis*	whole plant	0.0832
Compositae	*Artemisia schmidtiana* (= *A. sericea* var. *schmidtiana*)	whole plant	ND
Compositae	*Bidens tripartita*	whole plant	ND
Compositae	*Aster glehnii*	whole plant	ND
Compositae	*Bidens tripartita*	whole plant	0.0889
Compositae	*Chrysanthemum parthenium*	whole plant	ND
Compositae	*Agastache rugosa*	whole plant	ND
Compositae	*Arctium lappa*	whole plant	ND
Compositae	*Petasites japonicus* var. *giganteus*	whole plant	ND
Compositae	*Aster ageratoides* form. *yezoensis*	whole plant	ND
Compositae	*Helianthus tuberosus*	whole plant	ND
Compositae	*Hypochoeris radicata*	whole plant	ND
Compositae	*Solidago altissima*	whole plant	ND
Compositae	*Carpesium abrotanoides*	whole plant	ND
Compositae	*Cacalia hastata* var. *orientalis*	whole plant	ND
Compositae	*Senecio vulgaris*	whole plant	ND
Compositae	*Silybum marianum* (= *Carduus maranus*)	aerial part	ND
Compositae	*Galinsoga quadriradiata*	whole plant	ND
Compositae	*Papaver orientale*	whole plant	ND
Compositae	*Adenocaulon himalaicum*	whole plant	ND
Compositae	*Taraxacum officinale*	whole plant	ND
Compositae	*Sonchus oleraceus*	whole plant	ND
Compositae	*Inula japonica* (= *I. britannica* var. *japonica*)	whole plant	ND
Compositae	*Rudbeckia laciniata*	whole plant	ND
Compositae	*Solidago virga-aurea* var. *leiocarpa*	whole plant	ND
Compositae	*Atractylodes lancea*	whole plant	ND
Compositae	*Chrysanthemum leucanthemum*	whole plant	ND
Compositae	*Artemisia japonica*	whole plant	ND
Compositae	*Carpesium cernuum*	whole plant	ND
Compositae	*Xanthium strumarium*	whole plant	ND
Compositae	*Leibnitzia anandria*	whole plant	ND
Compositae	*Zinnia elegans*; *Z. peruviana*	whole plant	ND
Compositae	*Eupatorium chinense* var. *sachalinense*	whole plant	ND

Compositae	*Matricaria inodora*	whole plant	ND
Compositae	*Veronica americana*	whole plant	ND
Compositae	*Saussurea lappa*	whole plant	ND
Compositae	*Cirsium vulgare*	whole plant	ND
Compositae	*Lactuca scariola*	whole plant	ND
Compositae	*Farfugium japonicum*	whole plant	ND
Compositae	*Sonchus brachyotus* (= *S. arvensis* var. *ulginosus*)	whole plant	ND
Compositae	*Hieracium aurantiacum*	whole plant	ND
Compositae	*Lactuca* (*raddeana* var.) *elata*	whole plant	ND
Compositae	*Atractylodes japonica*	whole plant	ND
Compositae	*Echinops ritro*	whole plant	ND
Compositae	*Eupatorium rugosum*	whole plant	ND
Compositae	*Cosmos bipinnatus*	whole plant	ND
Compositae	*Breea setosa* var. *subulatum*	whole plant	ND
Campanulaceae	*Codonopsis lanceolata*	whole plant	ND
Campanulaceae	*Codonopsis ussuriensis*	whole plant	ND
Campanulaceae	*Adenophora triphylla* var. *japonica*	whole plant	ND
Menyanthaceae	*Menyanthes trifoliata*	whole plant	ND
Caprifoliaceae	*Sambucus sieboldiana* var. *miquelii*	branch・leaf	0.0801
Caprifoliaceae	*Lonicera morrowii* (= *Caprifolium morrowii*)	branch・leaf	ND
Caprifoliaceae	*Viburnum furcatum*	branch・leaf	ND
Caprifoliaceae	*Viburnum wrightii*	branch・leaf	0.0761
Caprifoliaceae	*Viburnum sargentii*	branch・leaf	ND
Caprifoliaceae	*Weigela hortensis*	branch・leaf	ND
Caprifoliaceae	*Weigela coraeensis*	branch・leaf	ND
Caprifoliaceae	*Lonicera caerulea* var. *amphyllocalyx*	branch・leaf	ND
Valerianaceae	*Patrinia scabiosaefolia*	whole plant	ND
Umbelliferae	*Cnidium officinale*	whole plant	0.0710
Umbelliferae	*Angelica keiskei*	branch・leaf	ND
Umbelliferae	*Angelica dahurica*	whole plant	ND
Umbelliferae	*Spuriopimpinella calycina*	whole plant	ND
Umbelliferae	*Cryptotaenia japonica*	whole plant	ND
Umbelliferae	*Aethusa cynapium*	whole plant	ND
Umbelliferae	*Conium maculatum*	whole plant	ND
Umbelliferae	*Aegopodium podagraria*	whole plant	0.0564
Umbelliferae	*Angelica ursina*	whole plant	ND
Umbelliferae	*Angelica edulis*	whole plant	ND
Umbelliferae	*Glehnia littoralis*	whole plant	ND
Umbelliferae	*Sanicula chinensis*	whole plant	ND

Umbelliferae	*Sium sisarum*	whole plant	ND
Umbelliferae	*Daucus carota*	whole plant	0.0807
Umbelliferae	*Bupleurum longiradiatum* subsp. *sachalinense* var. *elatius*	whole plant	ND
Umbelliferae	*Oenanthe javanica*	whole plant	ND
Umbelliferae	*Cicuta virosa*	whole plant	ND
Umbelliferae	*Angelica genuflexa*	whole plant	ND
Umbelliferae	*Anthriscus sylvestris*	whole plant	ND
Umbelliferae	*Conioselinum kamtschaticum*	whole plant	ND
Araliaceae	*Acanthopanax senticosus*	branch・leaf	ND
Araliaceae	*Aralia elata*	branch・leaf	ND
Araliaceae	*Kalopanax pictus*	branch・leaf	ND
Araliaceae	*Aralia cordata*	whole plant	ND
Araliaceae	*Acanthopanax divaricatus*	branch・leaf	ND
Araliaceae	*Acanthopanax sieboldianus*	stem・leaf	0.0580
Pinaceae	*Pinus banksiana*	branch・leaf	ND
Pinaceae	*Pinus koraiensis*	branch・leaf	0.0835
Pinaceae	*Pinus montana*	branch・leaf	0.0818
Pinaceae	*Picea pungens*	branch・leaf	ND
Pinaceae	*Pinus rigida*	branch・leaf	ND
Pinaceae	*Larix leptolepis*	branch・leaf	0.0600
Pinaceae	*Pinus strobus*	branch・leaf	0.0856
Pinaceae	*Pinus thunbergii*	branch・leaf	ND
Cupressaceae	*Thuja occidentalis*	branch・leaf	0.0933
Cupressaceae	*Platycladus orientalis ‘Semperaurea’*	branch・leaf・fruit	0.0965
Cupressaceae	*Juniperus chinensis* var. *procumbens*	branch・leaf	ND
Cupressaceae	*Chamaecyparis pisifera* var. *fllifera*	branch・leaf	ND
Taxodiaceae	*Metasequoia glyptostroboides*	branch・leaf	0.0660
Taxaceae	*Taxus cuspidata*	branch・leaf	ND
Ginkgoaceae	*Ginkgo biloba*	branch・leaf	ND
Ephedraceae	*Ephedra distachya*	whole plant	ND
Ophioglossaceae	*Botrychium robustum*	whole plant	ND
Equisetaceae	*Equisetum arvense*	whole plant	ND
Equisetaceae	*Equisetum hyemale*	whole plant	ND
Osmundaceae	*Osmunda japonica*	whole plant	0.0655
Dennstaedtiaceae	*Pteridium aquilinum* var. *latiusculum*	whole plant	ND
Aspleniaceae	*Asplenium scolopendrium*	whole plant	ND
Woodsiaceae	*Matteuccia orientalis*	whole plant	0.0534
Woodsiaceae	*Woodsia polystichoides*	whole plant	ND
Aspidiaceae	*Rumohra standishii*	whole plant	0.0125

Aspidiaceae	*Dryopteris crassirhizoma*	whole plant	0.0217
Aspidiaceae	*Polystichum tripteron*	whole plant	ND

Supplementary Table S2 Structures of compounds used 1st screening and the EC_{50} values by MSHTS system.

Compounds	No.<br>EC50 [μM]	Compounds	No.<br>EC50 [μM]	Compounds	No.<br>EC50 [μM]
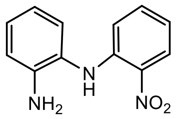	MO-001<br>215	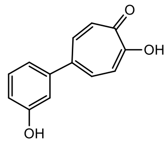	MO-012<br>209	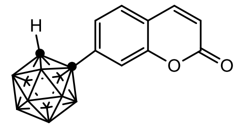	MO-023<br>ND
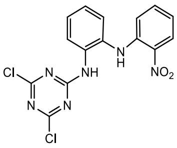	MO-002<br>ND	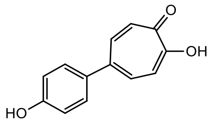	MO-013<br>ND	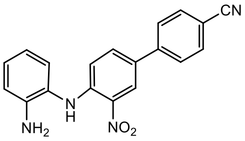	MO-024<br>ND
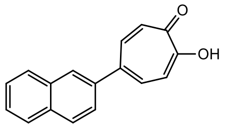	MO-003<br>317	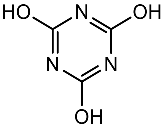	MO-014<br>ND	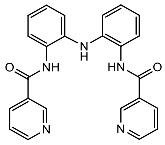	MO-025<br>ND
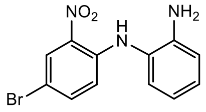	MO-004<br>369	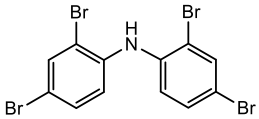	MO-015<br>ND	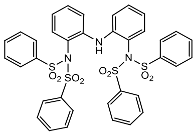	MO-026<br>ND
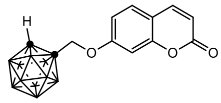	MO-005<br>ND	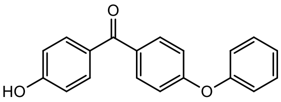	MO-016<br>501	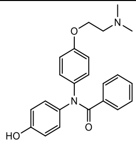	MO-027<br>ND
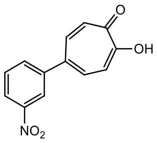	MO-006<br>ND	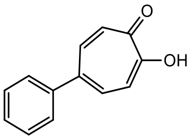	MO-017<br>ND	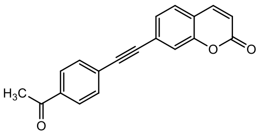	MO-028<br>ND
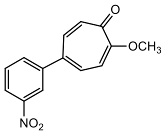	MO-007<br>144	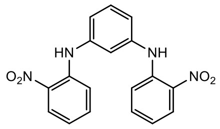	MO-018<br>ND	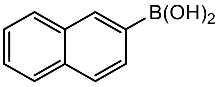	MO-029<br>ND
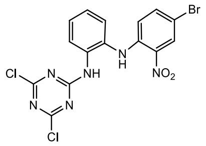	MO-008<br>11166	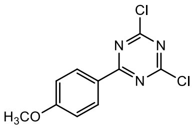	MO-019<br>ND	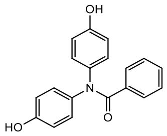	MO-030<br>ND
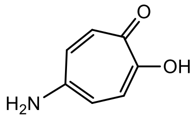	MO-009<br>19.1	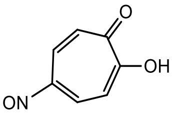	MO-020<br>154	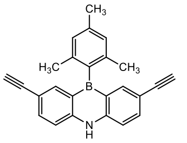	MO-031<br>ND
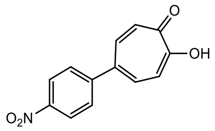	MO-010<br>52.5	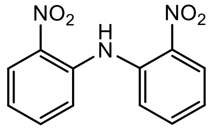	MO-021<br>ND	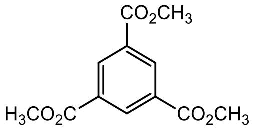	MO-032<br>ND
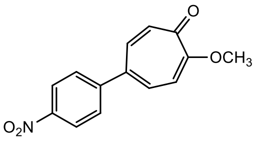	MO-011<br>200	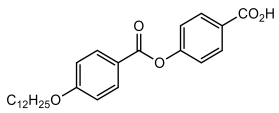	MO-022<br>ND	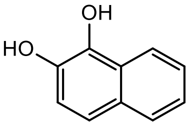	MO-033<br>ND

Compounds	No.<br>EC_{50} [µM]	Compounds	No.<br>EC_{50} [µM]	Compounds	No.<br>EC_{50} [µM]
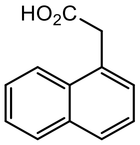	MO-034<br>ND	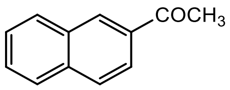	MO-045<br>ND	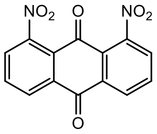	MO-056<br>ND
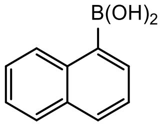	MO-035<br>ND	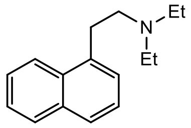	MO-046<br>ND	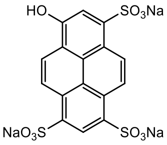	MO-057<br>ND
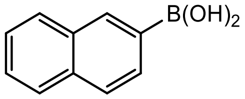	MO-036<br>ND	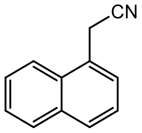	MO-047<br>ND	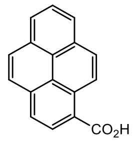	MO-058<br>ND
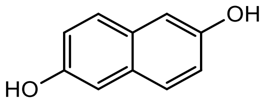	MO-037<br>ND	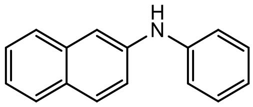	MO-048<br>ND	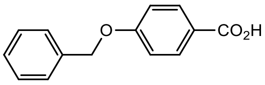	MO-059<br>ND
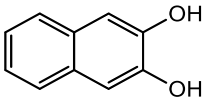	MO-038<br>31.4	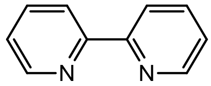	MO-049<br>ND	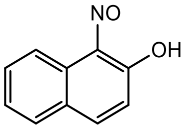	MO-060<br>ND
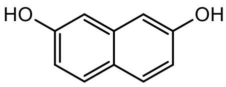	MO-039<br>ND	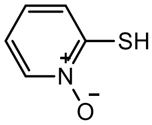	MO-050<br>ND	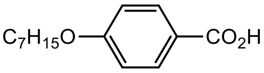	MO-061<br>ND
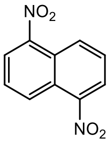	MO-040<br>ND	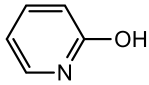	MO-051<br>ND	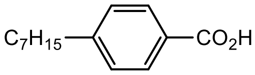	MO-062<br>ND
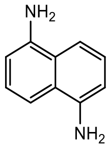	MO-041<br>ND	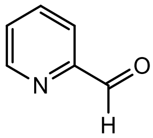	MO-052<br>ND	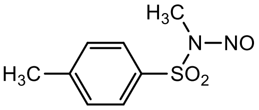	MO-063<br>ND
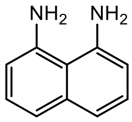	MO-042<br>ND	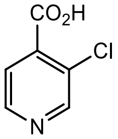	MO-053<br>ND	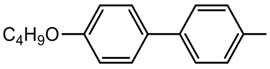	MO-064<br>ND
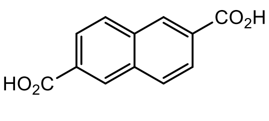	MO-043<br>ND	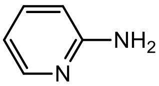	MO-054<br>ND	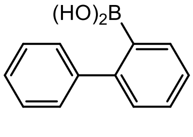	MO-065<br>ND
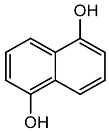	MO-044<br>ND	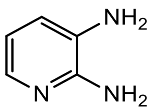	MO-055<br>ND	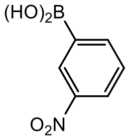	MO-066<br>ND

Compounds	No. EC50 [μM]	Compounds	No. EC50 [μM]	Compounds	No. EC50 [μM]
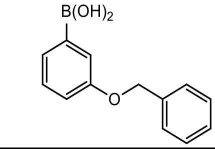	MO-067 ND	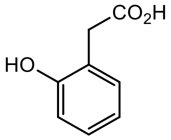	MO-078 ND	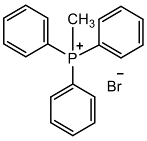	MO-090 ND
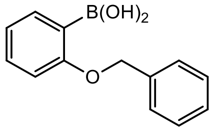	MO-068 ND	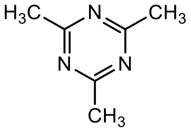	MO-079 ND	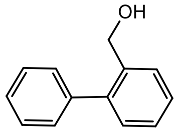	MO-091 ND
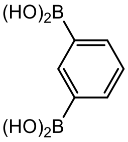	MO-069 ND	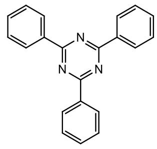	MO-080 ND	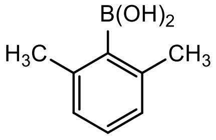	MO-092 ND
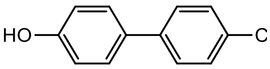	MO-070 ND	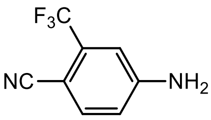	MO-082 ND	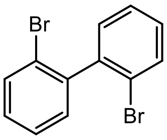	MO-094 ND
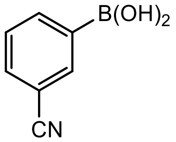	MO-071 ND	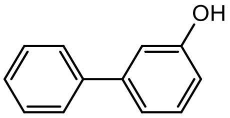	MO-083 ND	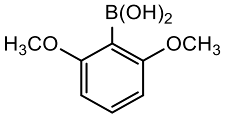	MO-095 ND
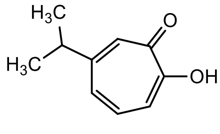	MO-072 ND	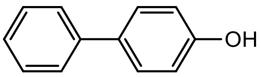	MO-084 ND	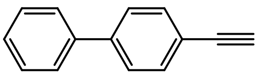	MO-096 ND
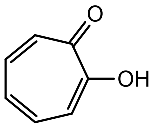	MO-073 ND	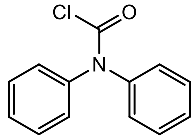	MO-085 ND	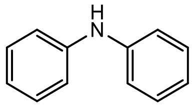	MO-098 ND
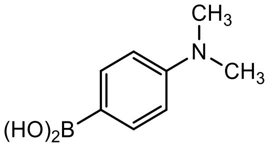	MO-074 53.1	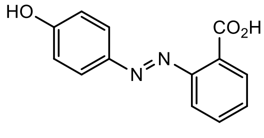	MO-086 ND	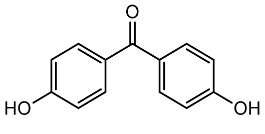	MO-099 ND
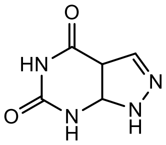	MO-075 ND	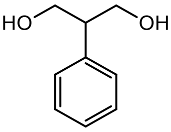	MO-087 ND	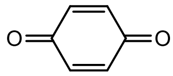	MO-100 56.0
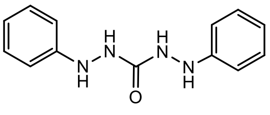	MO-076 ND	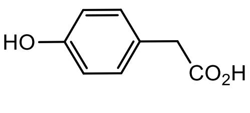	MO-088 ND	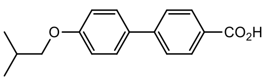	MO-101 ND
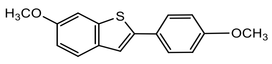	MO-077 ND	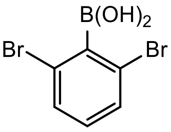	MO-089 ND		

MO-081, MO-093, and M0-097 are unused numbers.

Supplementary Table S3 Structures of compounds used in 2nd screening and the EC_{50} values by MSHTS system.

Compounds	No.<br>EC_{50} [μM]	Compounds	No.<br>EC_{50} [μM]	Compounds	No.<br>EC_{50} [μM]
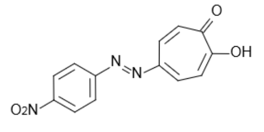	TR-001<br>258	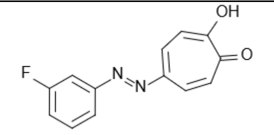	TR-013<br>257	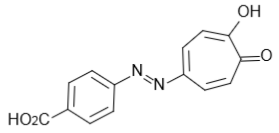	TR-026<br>ND
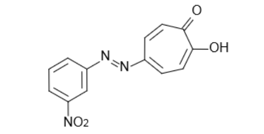	TR-002<br>232	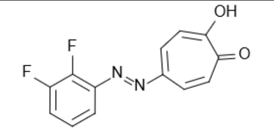	TR-014<br>44.1	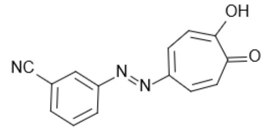	TR-027<br>38.7
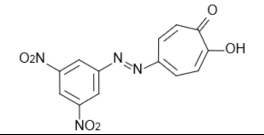	TR-003<br>6.60	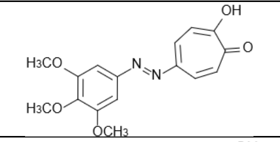	TR-015<br>192	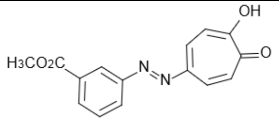	TR-028<br>347
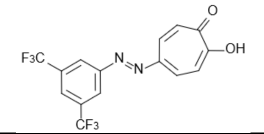	TR-004<br>ND	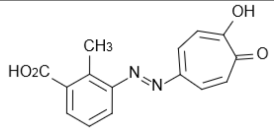	TR-016<br>386	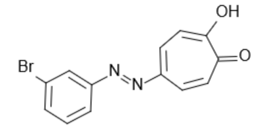	TR-029<br>489
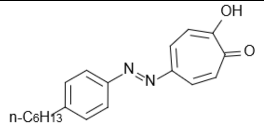	TR-005<br>66.6	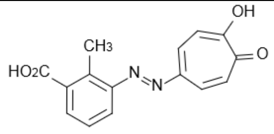	TR-017<br>1861	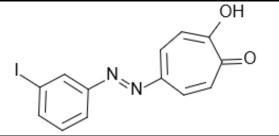	TR-030<br>416
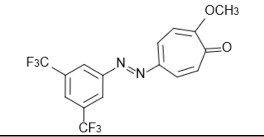	TR-006<br>ND	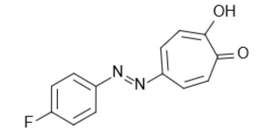	TR-018<br>541	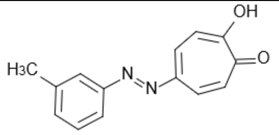	TR-031<br>108
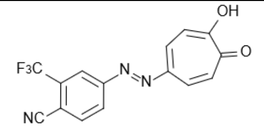	TR-007<br>13.9	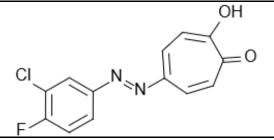	TR-019<br>260	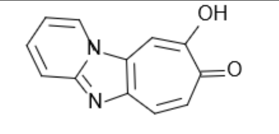	TR-033<br>234
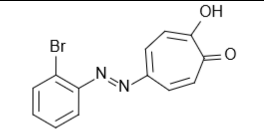	TR-008<br>43.5	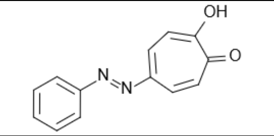	TR-020<br>372	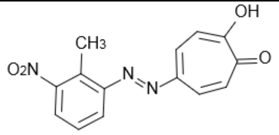	TR-034<br>176
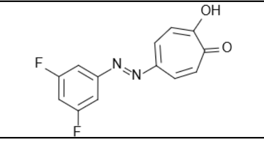	TR-009<br>71.3	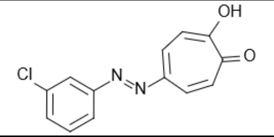	TR-021<br>914	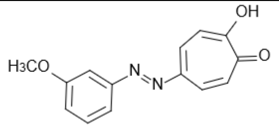	TR-035<br>126
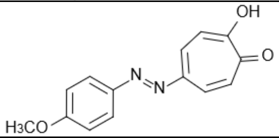	TR-010<br>216	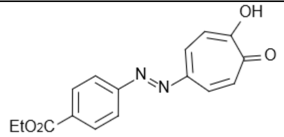	TR-022<br>3030	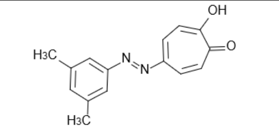	TR-036<br>82.7
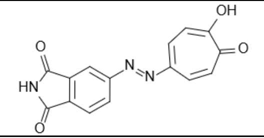	TR-011<br>16881	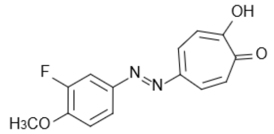	TR-023<br>838701	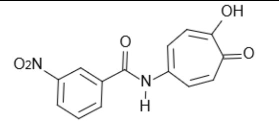	TR-038<br>ND
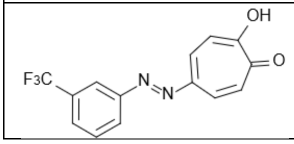	TR-012<br>82.1	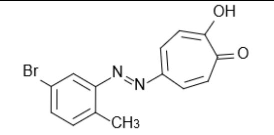	TR-024<br>ND	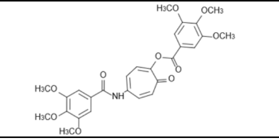	TR-101<br>ND

TR-025, TR-032, TR-37, and TR-039–TR-100 are unused numbers.

Supplementary Table S4 List of EC_{50} values of tropolone derivatives determined by the MSTHS system and ThT assay. Compounds are listed in descending order of activity by the MSHTS system.

No.	MSHTS [μM]	ThT [μM]
TR-003	6.6	18.4
TR-007	13.9	4.64
MO-009	19.1	87.6
TR-027	38.7	83
TR-008	43.5	5.95
TR-014	44.1	53.8
MO-010	52.5	144
TR-005	66.6	274
TR-009	71.3	20.1
TR-012	82.1	55.6
MO-007	144	ND
MO-020	154	152
MO-011	200	9193
MO-012	209	507
MO-003	261	ND
MO-006	ND	75
MO-073	ND	1611

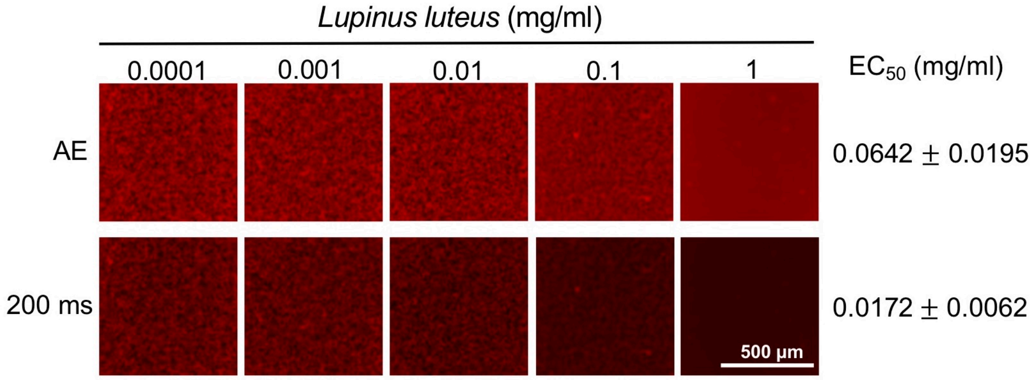

Supplementary Fig. S1 The EC_{50} values of crude extract were affected by the inner filter effect in the high concentration sample. Various concentrations of *Lupinus luteus* extract, which is a typical crude extract, were incubated with 30 μM Aβ and 30 nM QD_{655}-labeled Aβ in a 1536-well plate according to our previous reports[1,5]. Fluorescence micrographs show Aβ aggregation captured by auto exposure (AE) or 200 ms exposure. The means and SDs of EC_{50} values (n = 3) show that the inner filter effect by crude extract affected the EC_{50} value.

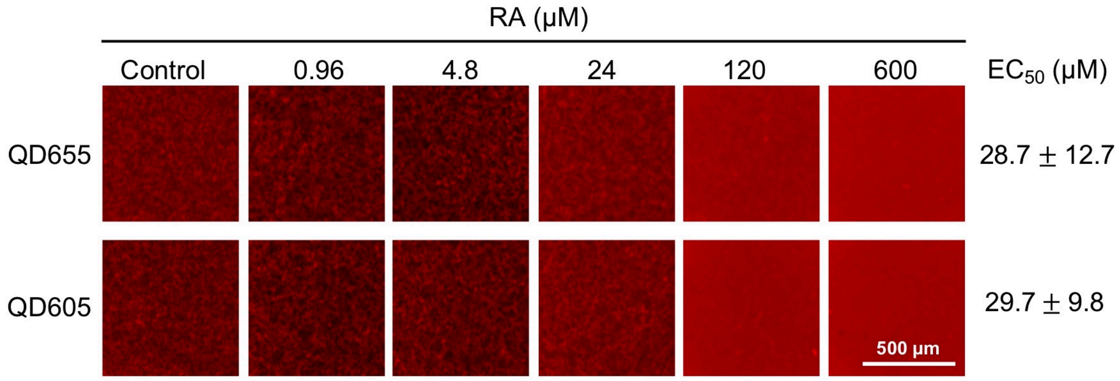

Supplementary Fig. S2 The difference in Qdot types did not affect EC_{50} values. Various concentrations of RA were incubated with 30 µM Aβ and 30 nM QD_{655}-labeled Aβ or QD_{605}-labeled Aβ in a 1536-well plate according to our previous reports[1,5]. Fluorescence micrographs show that Aβ aggregation with QD_{655}-labeled Aβ or QD_{605}-labeled Aβ were inhibited in the presence of 600 and 120 µM RA. The means and SDs of EC_{50} values (n = 3) show that the difference between QD_{655} and QD_{605} did not affect the EC_{50} value.

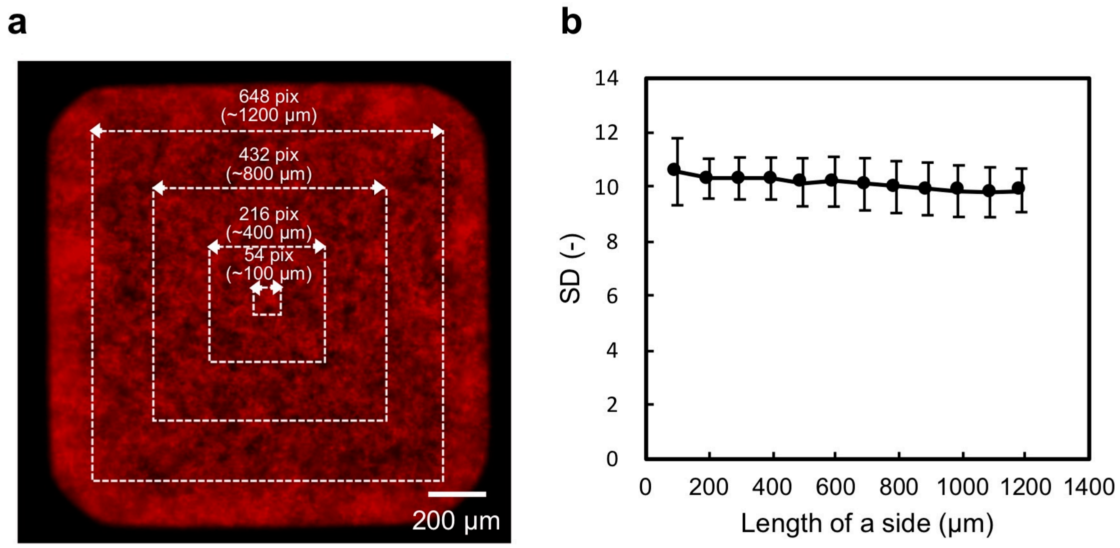

Supplementary Fig. S3 Relation between size of imaging area and SD value. 25 µM Aβ and 25 nM QDAβ were incubated in a 1536-well plate for 24 h at 37 °C, and wells were observed by fluorescence microscopy. **a**, Fluorescence microscopic images of typical Aβ aggregate without inhibitors. **b**, Average SD values determined from various imaging areas in central well region of 6 wells. Since 54 pixels is approximately 100 µm in this imaging system, we gradually increased the imaging area based on 54 pixels. The results showed that there was no significant difference in the SD value in the range of 100 to 1200 µm at the center of the well. On the other hand, the thickness of the aggregate tended to increase at the place of about a few hundred µm from the wall of the well. Since the out-of-focus aggregates affect the SD value[1], we decided to measure the SD value in the region of 800 × 800 µm (432 × 432 pixels) where the aggregates would not reliably contain thick regions.

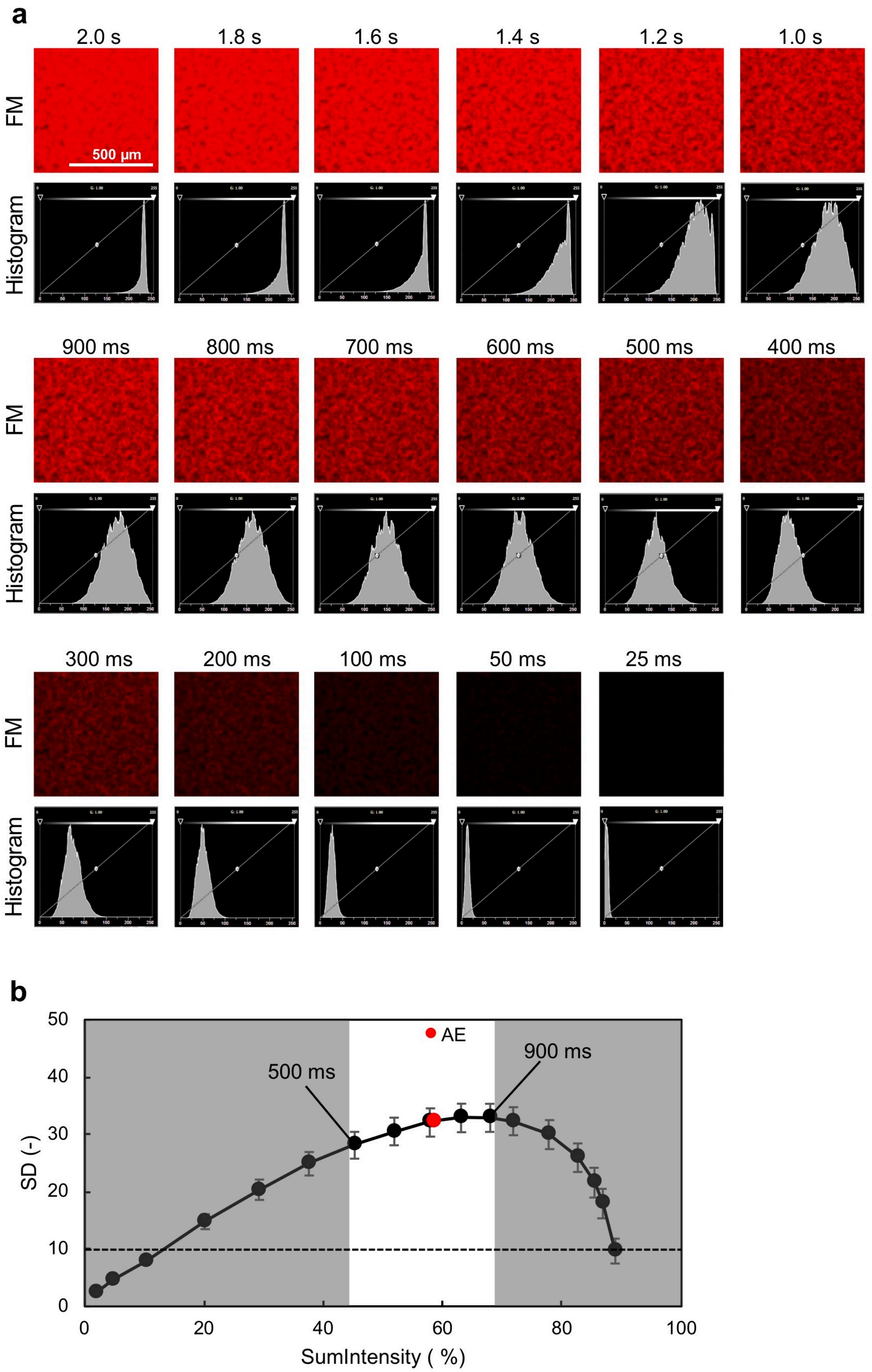

Supplementary Fig. S4 Examination of exposure time. 25 μM Aβ and 25 nM QDAβ were incubated in a 1536-well plate for 24 h at 37 °C, and wells were observed by fluorescence microscopy. **a**, Fluorescence micrographs (top) were taken at various exposure periods (2.0 s – 25

ms) and the histograms of fluorescence intensities of each pixel were plotted (bottom). **b**, The horizontal axis indicates the sum intensity of all pixels calculated from each image while the vertical axis indicates the SD value of brightness of each pixel. The SD value showed a relative maximum value between 500 and 900 ms when the histogram showed a normal distribution (white zone). The red symbol shows data when the target maximum light intensity of the camera setting was set at 50%, and the broken line indicates the mean SD values of brightness of each pixel before aggregation.

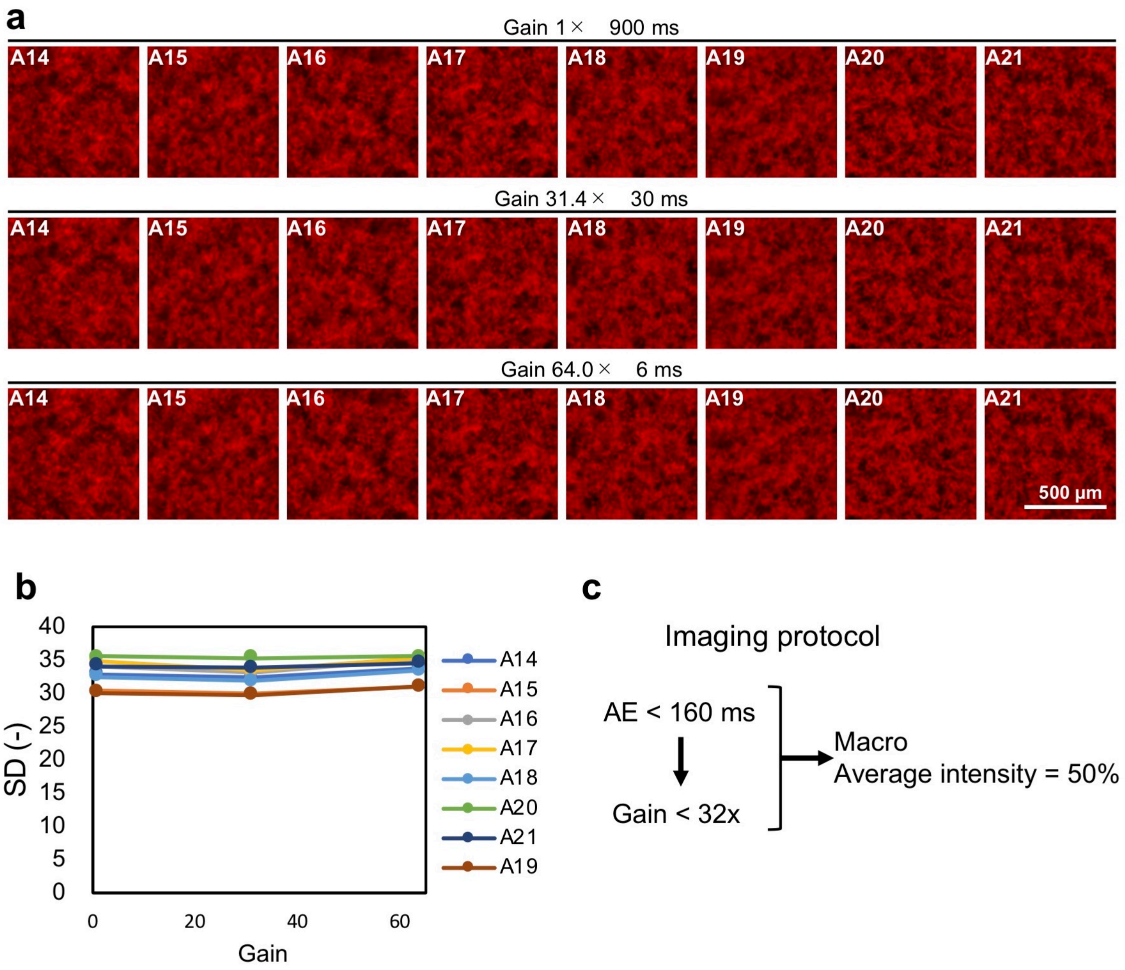

Supplementary Fig. S5 Examination of camera gain. 25 μM Aβ and 25 nM QDAβ were incubated in a 1536-well plate for 24 h at 37 °C, and wells were observed by fluorescence microscopy. **a**, Fluorescence microscopic images of 8 different wells observed under different gains and exposure conditions. The white character on the upper left of each image indicates the position of the well. **b**, Relation between camera gains and SD values of fluorescence intensity of each pixel. **c**, Imaging protocol. Auto exposure up to 160 ms and then the camera gain was increased to 32. The obtained image was adjusted by a macro program so that average intensity became 50%, and the SD value of fluorescence intensity of each pixel was determined.

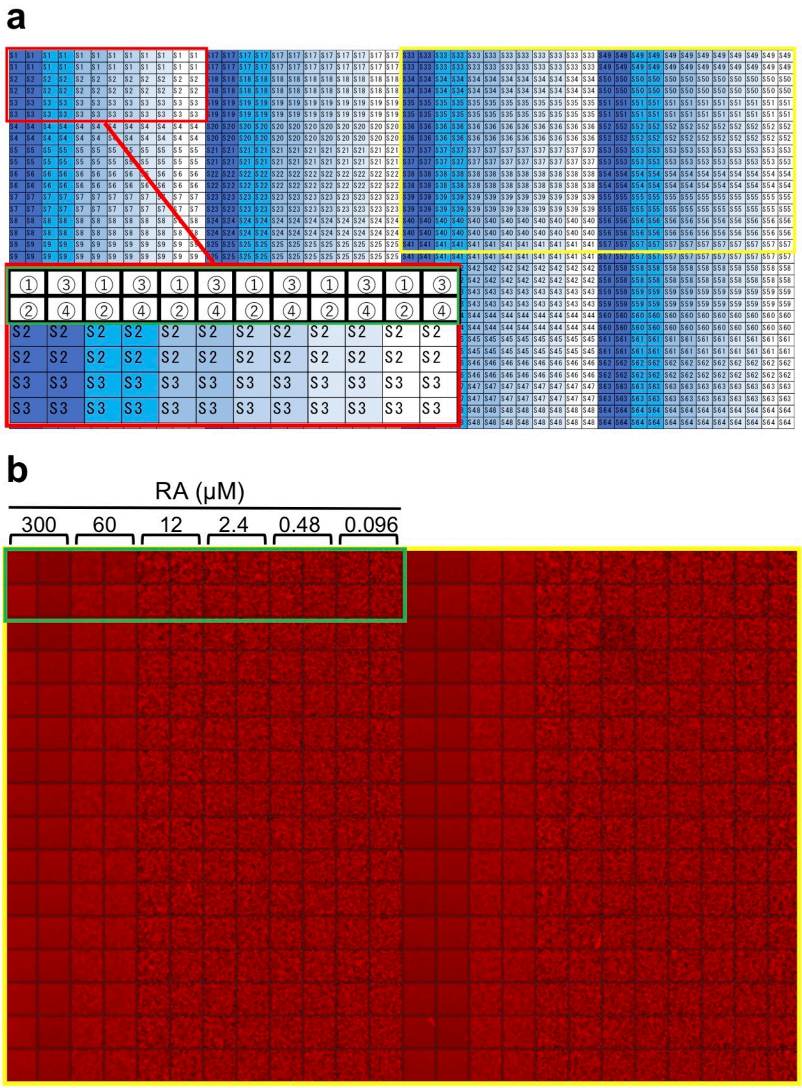

Supplementary Fig. S6 Automated MSHTS system. **a**, dilution series of each sample in a 1536-well plate. The enlarged red frame shows the concentration series of three samples (S1–S3). Each sample was diluted 5-times by a 5- or 10-fold dilution series, and injected into a 1536-well plate in the order of 1 to 4. Consequently, the single 1536-well plate can accommodate the analysis of 64 samples (n=4). **b**, Positive control experiment by RA. 25 μM Aβ and 25 nM QDAβ were incubated with 300, 60, 12, 2.4, 0.48, or 0.096 μM RA in a 1536-well plate for 24 h at 37 °C, and the center of each well was observed by fluorescence microscopy. This image summarizes the micrographs of the yellow frame area in a. Green frame shows one concentration series from 300 to 0.096 μM of RA.

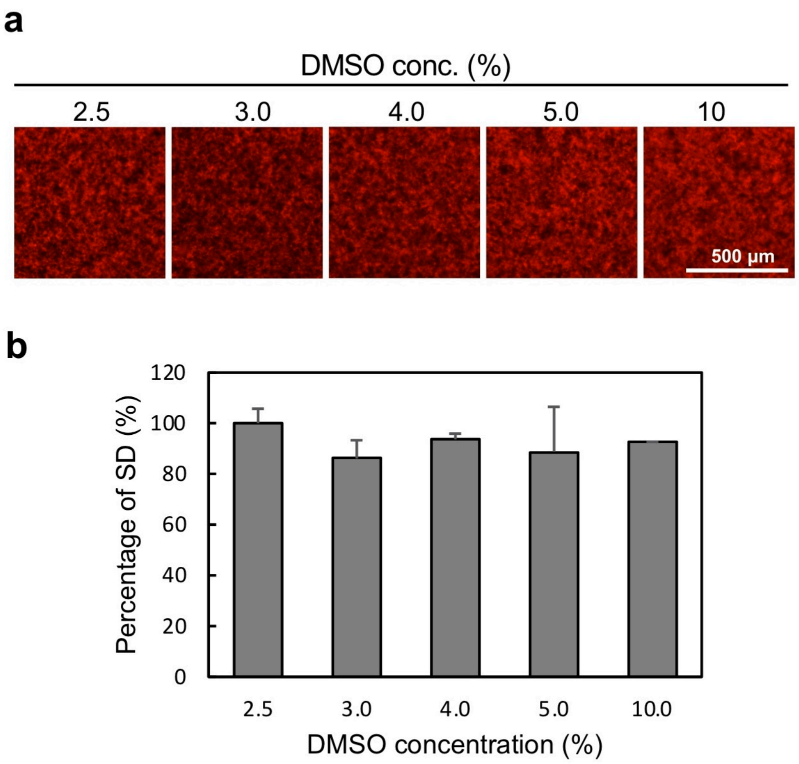

Supplementary Fig. S7 Effect of DMSO concentration for Aβ aggregation. 25 µM Aβ and 25 nM QDAβ containing various concentrations of DMSO were incubated in a 1536-well plate for 24 h at 37 °C, and wells were observed by fluorescence microscopy. **a**, Fluorescence microscopic images of Aβ aggregates containing 2.5, 3.0, 4.0, 5.0, and 10% DMSO. 2.5% DMSO was brought from Aβ solution because 1 mM Aβ in DMSO is diluted to a final concentration of 25 µM Aβ. **b**, Percentage of SD values of fluorescence intensity under each DMSO condition (n=3). There was no significant differences in these conditions (2.5–10% DMSO concentration). In this study, the influence of DMSO will be negligible since the concentration of DMSO brought from the plant and compound libraries are at most 0.1% and 1.5%, respectively.

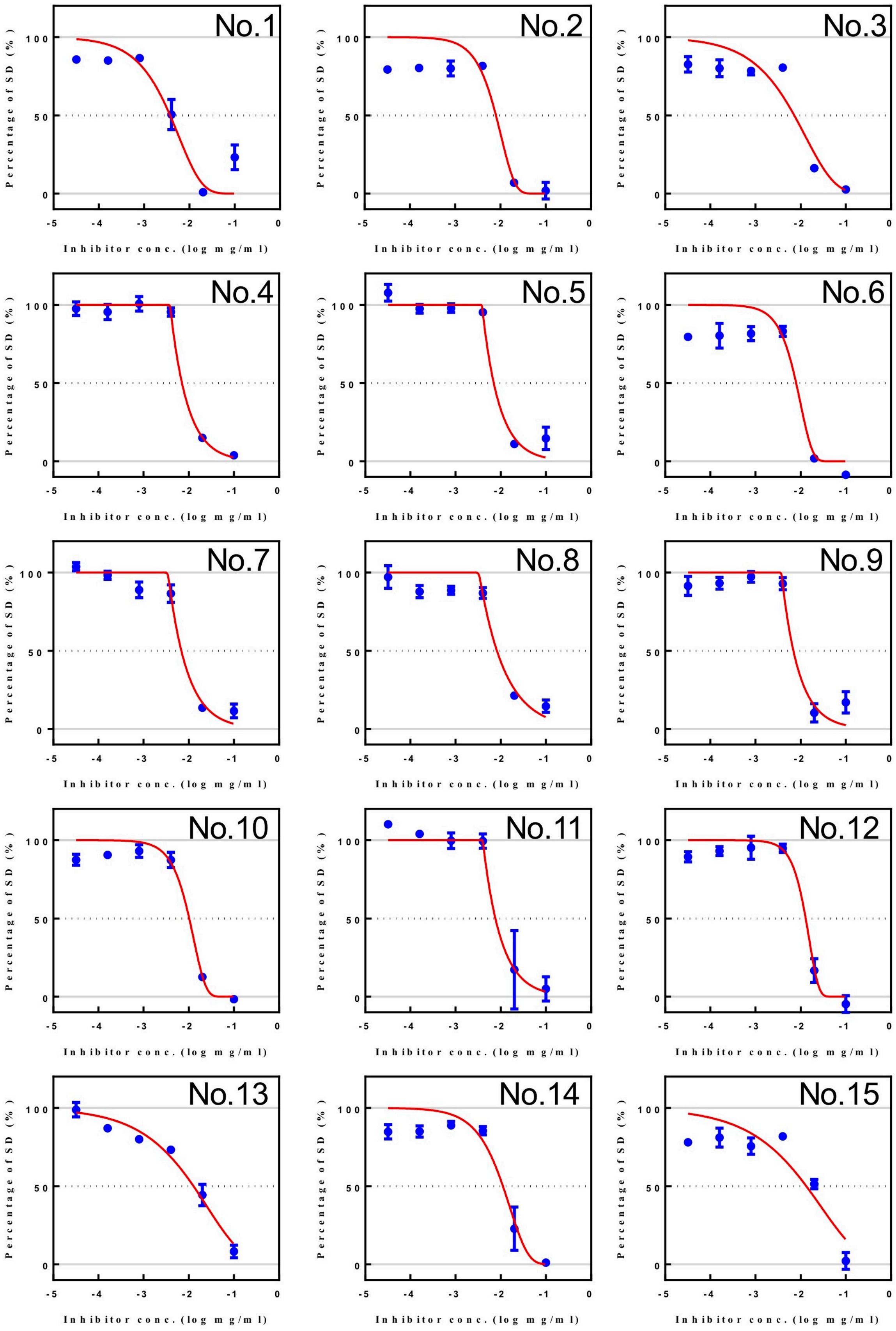

Supplementary Fig. S8-1 Inhibition curves of crude plant extracts showing high Aβ aggregation inhibitory activity (No. 1–15). The number shows the order of activity in Fig. 2a.

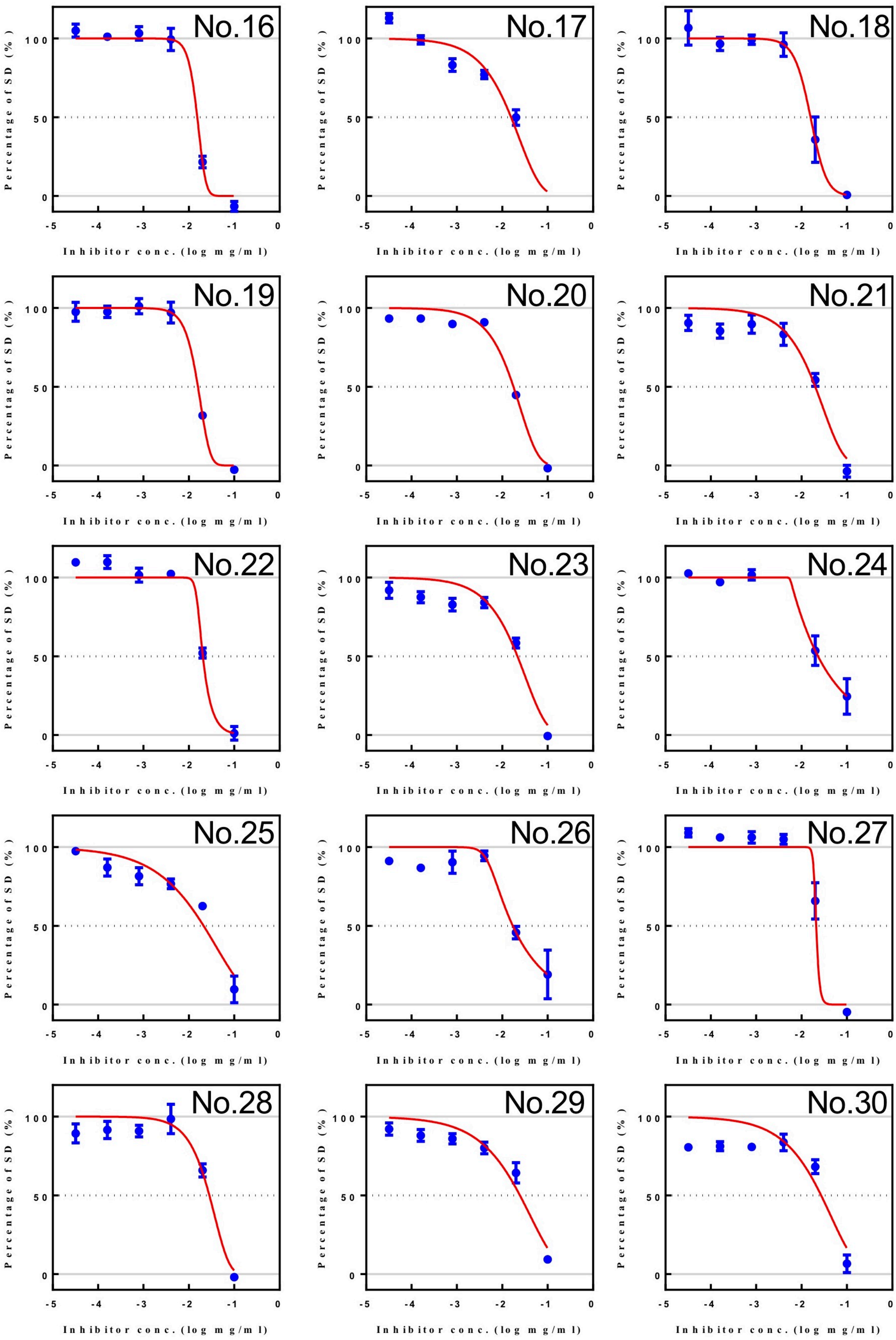

Supplementary Fig. S8-2 Inhibition curves of crude plant extracts showing high Aβ aggregation inhibitory activity (No. 16–30). The number shows the order of activity in Fig. 2a.

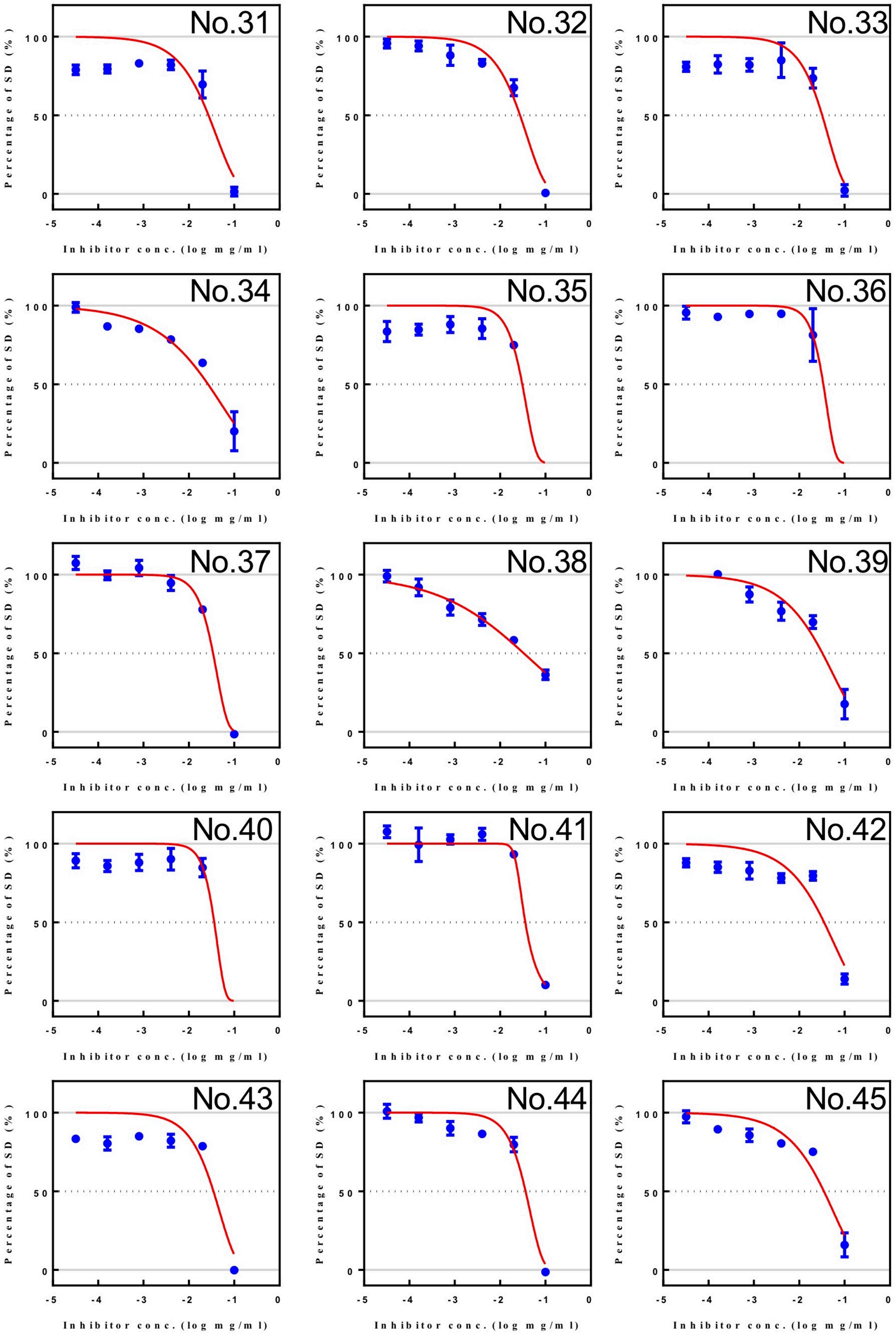

Supplementary Fig. S8-3 Inhibition curves of crude plant extracts showing high Aβ aggregation inhibitory activity (No. 31–45). The number shows the order of activity in Fig. 2a.

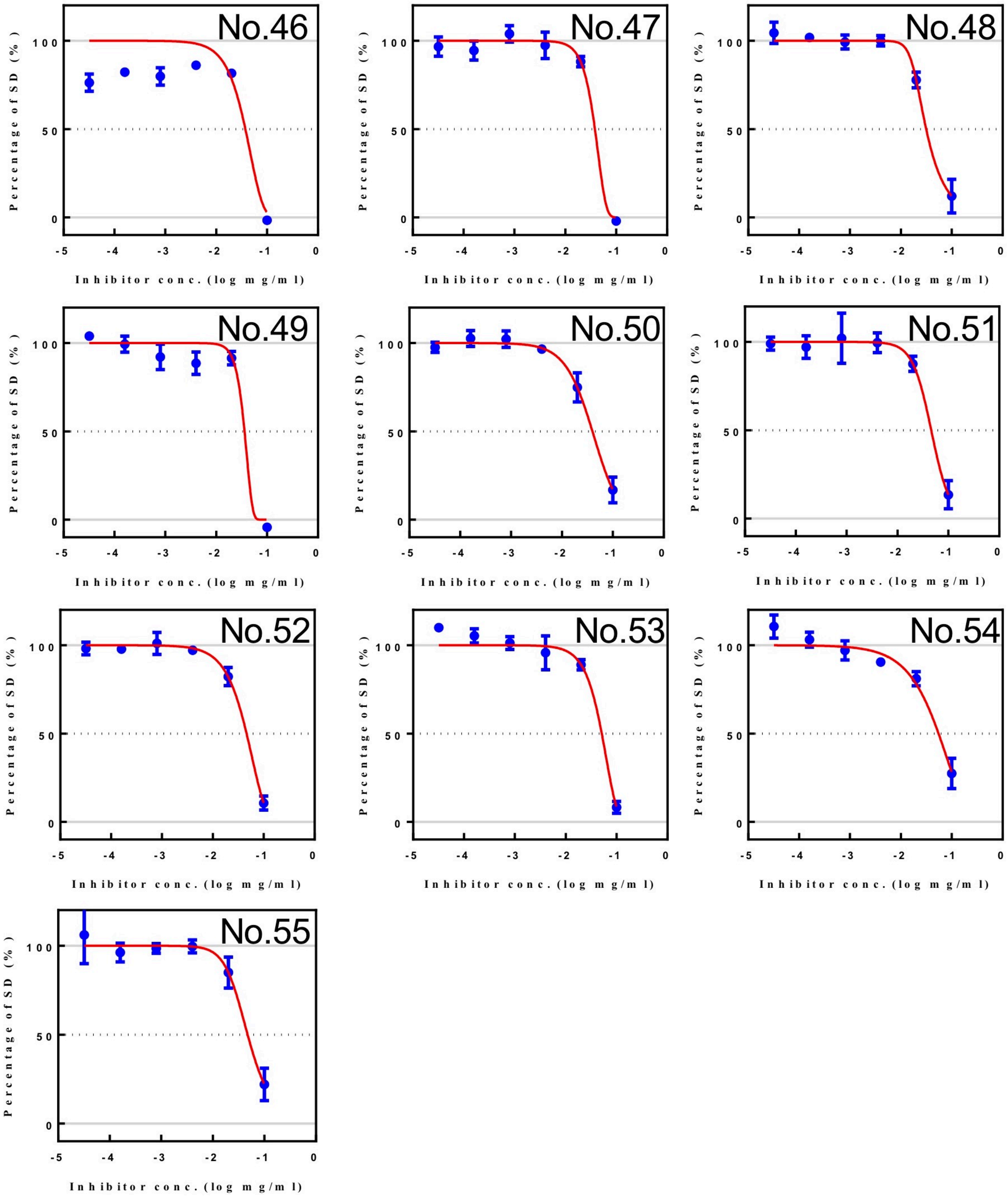

Supplementary Fig. S8-4 Inhibition curves of crude plant extracts showing high Aβ aggregation inhibitory activity (No. 46–55). The number shows the order of activity in Fig. 2a.

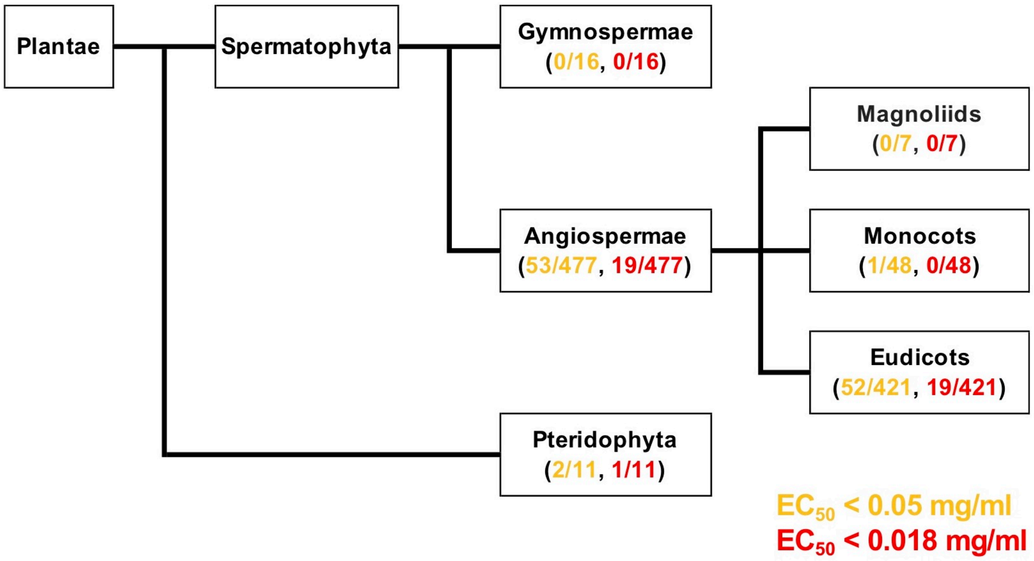

Supplementary Fig. S9 Aβ aggregation inhibitory activity based on plant classification. Numbers in parentheses indicate the number of plant extracts with high activity. Yellow and red numbers showed $EC_{50} < 0.05$ mg/ml and $EC_{50} < 0.018$ mg/ml, respectively.

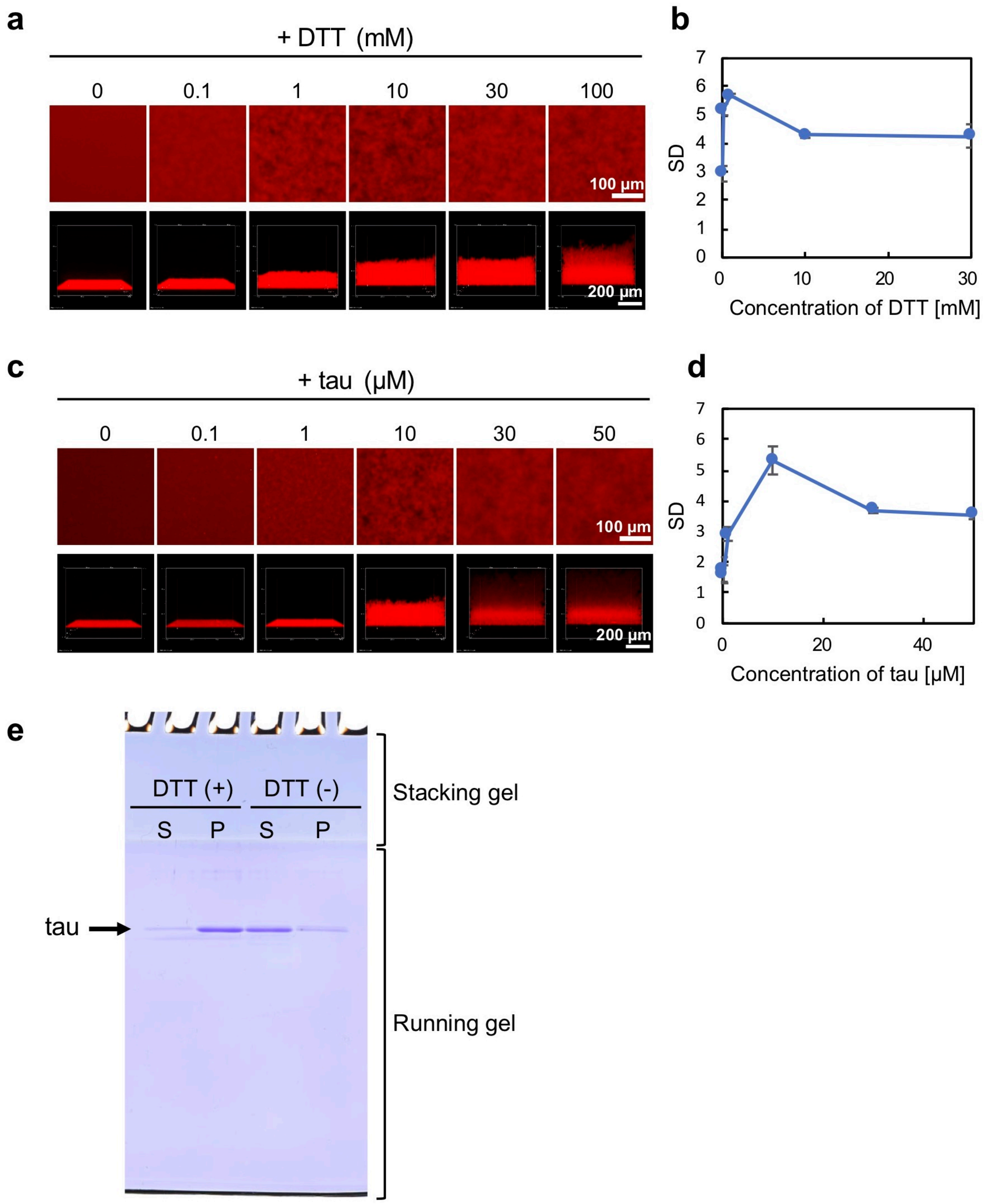

Supplementary Fig. S10 Examination of tau aggregation status. **a**, Relation between DTT concentration and tau aggregation. 10 μM tau, 50 nM QDTau, and 10 μM heparin were incubated with various concentrations of DTT in a 1536-well plate for 24 h at 37 °C, and observed by fluorescence microscopy (top) and confocal microscopy (bottom). **b**, SD values of fluorescence intensities of each pixel were determined from 2D images by fluorescence microscopy (a, top), and plotted against the concentration of added DTT. **c**, Concentration-dependent tau aggregation. Various concentrations of tau, 50 nM QDTau, and 10 μM heparin were incubated with 10 mM DTT in a 1536-well plate for 24 h at 37 °C, and observed by fluorescence microscopy (top) and confocal microscopy (bottom). **d**, SD values of fluorescence intensities of each pixel were determined from

2D images by fluorescence microscopy (c, top), and plotted against the concentration of added tau. **e**, Sedimentation assay of tau. 10 μM tau, 50 nM QDTau, and 10 μM heparin were incubated in the presence (+) or absence (-) of 10 mM DTT in a microtube for 24 h at 37 °C and centrifuged at 386,000 ×*g* for 15 min at 4 °C. Supernatants (S) and pellets (P) were electrophoresed on a 15% SDS-polyacrylamide gel. Full-length gel containing stacking gel and running gel is shown.

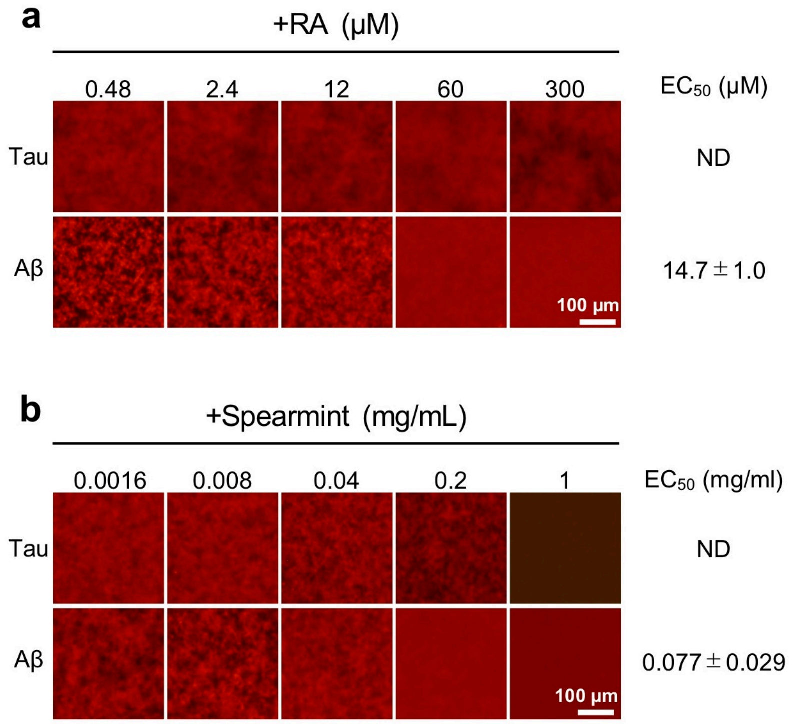

Supplementary Fig. S11 Inhibition of tau aggregation by RA (a) and spearmint extract (b). 10 μM tau, 50 nM QDTau, 10 μM heparin, 10 mM DTT were incubated with various concentrations of RA and spearmint extract, which showed highest activity among 52 spice extracts[1], in a 1536-well plate for 24 h at 37 °C and observed by fluorescence microscopy. EC_{50} and the SD values were determined from the micrographs of three independent wells.

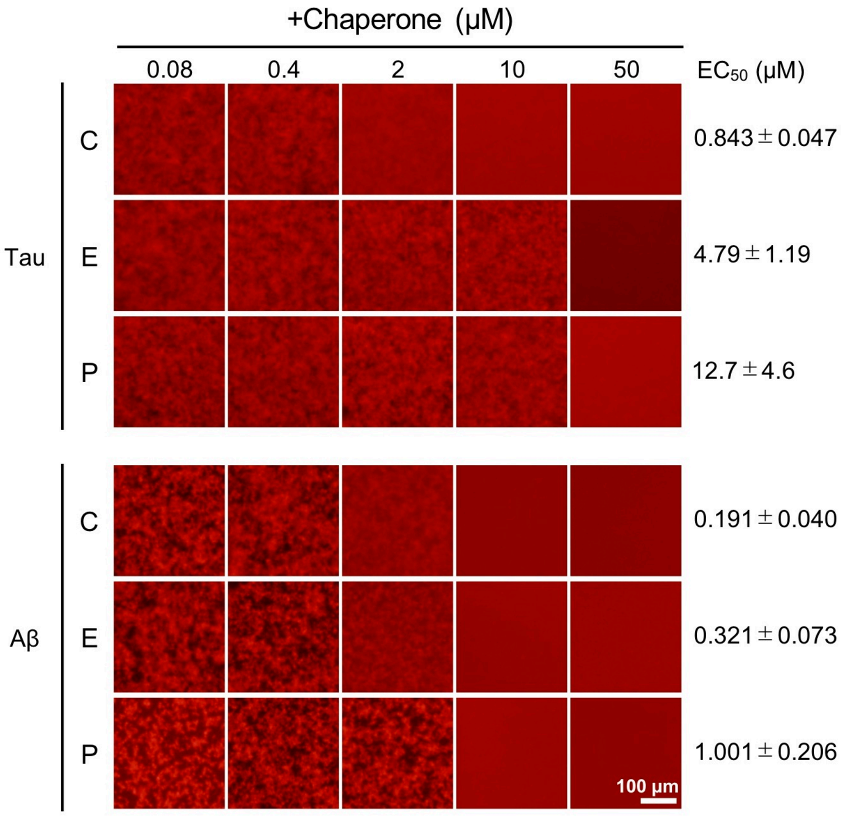

Supplementary Fig. S12 Inhibition of tau and Aβ aggregation by chaperones. 10 μM tau, 50 nM QDTau, 10 μM heparin, 10 mM DTT were incubated with various concentrations of chaperones in a 1536-well plate for 24 h at 37 °C and observed by fluorescence microscopy. 30 μM Aβ, 30 nM QDAβ were incubated with various concentrations of chaperones in a 1536-well plate for 24 h at 37 °C and observed by fluorescence microscopy. C, E, and P indicate CRT, ERp57, and PDI, respectively. EC_{50} and the SD values were determined from the micrographs of three independent wells.

References

1 Ishigaki, Y. *et al.* A microliter-scale high-throughput screening system with quantum-dot nanoprobes for amyloid-beta aggregation inhibitors. *PLoS One* **8**, e72992, doi:10.1371/journal.pone.0072992 (2013).

2 Tokuraku, K. *et al.* The number of repeat sequences in microtubule-associated protein 4 affects the microtubule surface properties. *J. Biol. Chem.* **278**, 29609-29618, doi:10.1074/jbc.M302186200 (2003).

3 Lowry, O. H., Rosebrough, N. J., Farr, A. L. & Randall, R. J. Protein measurement with the Folin phenol reagent. *J. Biol. Chem.* **193**, 265-275 (1951).

4 Laemmli, U. K. Cleavage of structural proteins during the assembly of the head of bacteriophage T4. *Nature* **227**, 680-685 (1970).

5 Ogara, T., Takahashi, T., Yasui, H., Uwai, K. & Tokuraku, K. Evaluation of the effects of amyloid beta aggregation from seaweed extracts by a microliter-scale high-throughput screening system with a quantum dot nanoprobe. *J. Biosci. Bioeng.* **120**, 45-50, doi:10.1016/j.jbiosc.2014.11.018 (2015).
